# Regional variation of the cortical and trabecular bone material properties in the rabbit skull

**DOI:** 10.1371/journal.pone.0298621

**Published:** 2024-02-27

**Authors:** Linje Wang, Carlo Meloro, Michael J. Fagan, Roger W. P. Kissane, Karl T. Bates, Graham N. Askew, Peter J. Watson

**Affiliations:** 1 Structural Biomechanics, Department of Civil and Environmental Engineering, Imperial College London, London, United Kingdom; 2 School of Engineering, University of Hull, Hull, United Kingdom; 3 Research Centre in Evolutionary Anthropology and Palaeoecology, School of Biological and Environmental Sciences, Liverpool John Moores University, Liverpool, United Kingdom; 4 Department of Musculoskeletal & Ageing Science, Institute of Life Course and Medical Sciences, University of Liverpool, Liverpool, United Kingdom; 5 School of Biomedical Sciences, University of Leeds, Leeds, United Kingdom; 6 Institute of Medical and Biological Engineering, School of Mechanical Engineering, University of Leeds, Leeds, United Kingdom; University of Gothenburg: Goteborgs Universitet, SWEDEN

## Abstract

The material properties of some bones are known to vary with anatomical location, orientation and position within the bone (e.g., cortical and trabecular bone). Details of the heterogeneity and anisotropy of bone is an important consideration for biomechanical studies that apply techniques such as finite element analysis, as the outcomes will be influenced by the choice of material properties used. Datasets detailing the regional variation of material properties in the bones of the skull are sparse, leaving many finite element analyses of skulls no choice but to employ homogeneous, isotropic material properties, often using data from a different species to the one under investigation. Due to the growing significance of investigating the cranial biomechanics of the rabbit in basic science and clinical research, this study used nanoindentation to measure the elastic modulus of cortical and trabecular bone throughout the skull. The elastic moduli of cortical bone measured in the mediolateral and ventrodorsal direction were found to decrease posteriorly through the skull, while it was evenly distributed when measured in the anteroposterior direction. Furthermore, statistical tests showed that the variation of elastic moduli between separate regions (anterior, middle and posterior) of the skull were significantly different in cortical bone, but was not in trabecular bone. Elastic moduli measured in different orthotropic planes were also significantly different, with the moduli measured in the mediolateral direction consistently lower than that measured in either the anteroposterior or ventrodorsal direction. These findings demonstrate the significance of regional and directional variation in cortical bone elastic modulus, and therefore material properties in finite element models of the skull, particularly those of the rabbit, should consider the heterogeneous and orthotropic properties of skull bone when possible.

## Introduction

The material properties of bone are known to be dependent on location within a bone [1–3]. Material properties have even been shown to vary between different structures of a bone (i.e. cortical and trabecular bone) [4–6], although this is not consistently observed [7], thus highlighting the complex relationship between material properties (such as elastic modulus) and anatomical location. This complexity is increased further when considering the often anisotropic nature of bone [8, 9].

The material properties of bone are an essential input for computational biomechanical modelling of anatomical structures, particularly finite element analysis (FEA), which is a non-invasive technique now commonly used to estimate the stresses and strains in soft and hard tissues under physiological loading. The skull is a good example of an anatomical structure that is frequently modelled through FEA in a wide range of taxa [10–19].

Some studies have measured the material properties of bones in the mammalian skull using a range of techniques, such as indentation [20, 21], compression testing [22] and ultrasonic waves [23–25]. These studies have shown that the mammalian skull displays regional variation and anisotropy of bone material properties. FEA studies of the skull have highlighted how material properties can influence the predicted strains in bone, particularly when representing the heterogenous [26] and orthotropic [27, 28] nature of bone, in comparison to assuming the bone acts in a homogenous, isotropic manner.

Datasets detailing the variation of material properties with respect to location and anisotropy in the skull are still sparse, and are limited to only a small number of species. Therefore, the use of homogeneous, isotropic material properties in FEA studies of the skull is often unavoidable, and prevails across the literature [10–19]. Furthermore, sometimes this results in FEA studies using data from a different species to the one under investigation [29–31].

The rabbit *(Oryctolagus cuniculus)* is one of many species yet to be investigated in terms of bone heterogeneity and anisotropy throughout the skull, and how sensitive FEA modelling is to potential regional variations in material properties. This is surprising considering the biomechanics of the rabbit on skull is of interest to numerous scientific fields: firstly, to investigate form-function relationships, such as the biomechanical relevance of the fenestrated rostrum; secondly, understanding the healthy rabbit masticatory system is a prerequisite for several hypotheses of dental disease [32–34], one of the most commonly reported diseases in the rabbit; and thirdly, to develop computational biomechanical models of the rabbit that can simulate the healthy rabbit masticatory system [31, 35], which have a potential application in the replacement, refinement, and reduction (3Rs) of experiments in biomedical and veterinary research. Therefore, understanding the regional variation of bone material properties in rabbit skull has a direct application in such studies, and would also be of intertest to studies of other lagomorphs.

Bone in the rabbit skull is known to display varying levels of adaption in response to elevated masticatory loading in terms of both regional variation, and responses at different levels of bony architecture (cortical thickness and biomineralisation) at the same site [36–38]. However, studies have also reported that the bone in the neurocranium is unaffected by increased mastication loads [37, 38]. Although this suggests the presence of a complex link between bone material properties and mastication in the rabbit, these studies were limited to a few sites within the skull, therefore the heterogeneity and anisotropy across the whole structure is yet to be reported. Therefore, this study aimed to measure the elastic modulus by the application of nanoindentation to test the following hypotheses:

**Hypothesis 1:** The elastic modulus of bone in the rabbit skull will not differ significantly between separate regions of the skull;
**Hypothesis 2:** The elastic modulus of bone in the rabbit skull will not differ significantly between individuals fed similar diets;
**Hypothesis 3:** The elastic modulus of bone in the rabbit skull will not differ significantly between orthotropic directions.

A dataset capable of testing these hypotheses will be valuable to inform future rabbit skull FEA studies of suitable bone material properties, and enable such studies to investigate the complexity of material modelling required to achieve accurate predictions using FEA.

## Methods

All experimental procedures were performed in accordance with the UK animal scientific procedures act (1986) and approved by the University of Liverpool Animal Welfare and Ethical Review Committees. This work conforms to the ethical requirements outlined by the journal, and presented in accordance with guidelines for animal work [39]. Three male New Zealand White rabbits (Envigo) (2.71 ± 0.09 kg, 13–14 weeks of age, 8.77 ± 0.14 cm mean skull length) were used in this study. Animals were housed under a 12 hour light:dark cycle at 21 °C and had *ad libitum* access to food and water. Rabbits were culled with an overdose of pentobarbital.

**Fig 1:**
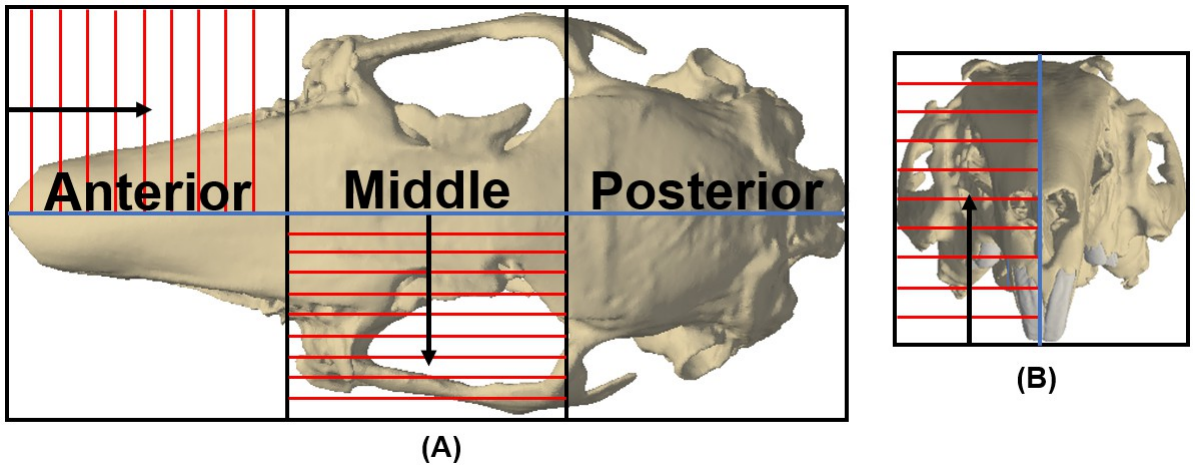
Sectioning of the rabbit skull into six separate regions. Regions were defined about the midline of the skull in the transverse and frontal plane (illustrated by the blue lines) with three roughly equally sized regions dividing the length of the skull into anterior, middle and posterior regions; thus creating an anterior, middle and posterior region on both sides of the skull. Material properties were measured on multiple slices throughout each region (example slices are indicated by the red lines). The direction of the slices was dependent on the direction in which a region was analysed, for example: a) shows the slicing of the anterior region in the anteroposterior (AP) direction, and slicing of the middle region in the mediolateral (ML) direction (direction of the slicing is shown by the black arrow); b) shows slicing in the ventrodorsal (VD) direction (direction of the slicing is shown by the black arrow).

The skulls of the three rabbits were sectioned into six separate regions (Fig 1) using a Buehler Isomet 5000 linear saw (Buehler, United States). Each region was defined about the midline of the skull separating the left and right sides, when viewed in the transverse and frontal plane. Three regions of roughly equal size were created on either side of the midline, thus creating anterior, middle and posterior regions for both the left and right sides of the skull (Fig 1). Three regions were used to allow easy potting and testing of the complete skull, as follows. Each region was set in Epo-Flo resin (MetPrep, United Kingdom), and either the anterior, medial or ventral surface of the region was polished on a Buehler Phoenix Beta grinder/polisher (Buehler, United States). Each region was then analysed using the following procedure: 1. the region was soaked in a phosphate-buffered saline (PBS) solution for 15 minutes, and then several indentations were made on the polished surface with a UNHT3 nanoindenter (Anton-Paar, Austria), using a Berkovich tip (chosen for its wide application in the indentation of bone [21, 40, 41]; 2. the region was then ground down by typically 1.5 mm using a Buehler Phoenix Beta grinder/polisher in a perpendicular direction to the polished surface (i.e. in either the anteroposterior [AP], mediolateral [ML] or ventrodorsal [VD] direction) (Fig 1), and the new ground surface was then repolished; 3. the region was re-soaked in PBS for 15 minutes, and more indentations were made on the newly polished surface. This procedure was repeated through the section under investigation, so that each region was analysed through numerous slices (Fig 1).

To test for variation in elastic modulus through the skull, and between different orthotropic directions, regions located on one side of the skull (i.e. the anterior, middle and posterior on the left side) were sliced in one orthogonal direction, whereas regions on the other side of the skull were sliced in an alternative direction. While it was not possible to test the same individual in all three orthogonal directions, the testing setup did enable: the elastic modulus in the ML direction to be compared between all individuals; and, the elastic modulus in the AP direction to be compared between Rabbits 1 and 2 (Fig 1; Table 1).

**Table 1:** The significance of variation in cortical bone elastic modulus within a region. Significance difference within a region was determined through either an ^a^ANOVA (p < 0.05) or ^b^Kruskal-Wallis (p < 0.05) test. The Homogeneity of Variance based on the median was used to determine an appropriate statistical method to test for significant differences between slices within a region; either a ^c^Tukey’s post hoc test, ^d^Dunn’s post hoc test or ^e^Dunnett’s T3 post hoc test. The direction in which a region was analysed is defined as: anteroposterior (AP), mediolateral (ML) and ventrodorsal (VD).

	Region / Slicing direction	No. of slices	No. of indents per slice	Average Elastic Modulus (GPa)	Significance between slices	
**Rabbit 1**	Anterior / AP	19	5–10	18.62 (3.60)	**p < 0.001** ^**a**^	Slice 1 < Slices 17-19^c^ Slice 17 > Slices 2 & 4^c^
	Middle / AP	28	4–13	17.94 (3.55)	**p < 0.021** ^**a**^	Slice 12 > Slice 18^c^
	Posterior / AP	16	3–13	16.32 (5.11)	**p < 0.001** ^**b**^	Slice 3 > Slice 15^d^ Slice 9 < Slices 1 & 3^d^
	Anterior / ML	5	2–10	11.14 (3.32)	**p < 0.001** ^**a**^	Slice 1 < Slices 2 & 4^e^
	Middle / ML	16	3–11	14.78 (4.13)	**p = 0.002** ^**a**^	Slice 1 < Slices 7 & 12^e^
	Posterior / ML	12	3–12	15.78 (4.74)	**p = 0.013** ^**a**^	Slice 1 < Slices 5 & 8^c^
**Rabbit 2**	Anterior / AP	19	3–10	18.55 (3.80)	**p = 0.003** ^**a**^	Slice 1 < Slices 14 & 17^c^ Slice 2 < Slice 17^c^
	Middle / AP	17	3–12	17.86 (3.36)	p = 0.115^a^	-
	Posterior / AP	19	2–12	17.96 (4.73)	**p = 0.017** ^**a**^	Slice 17 < Slices 3, 4, 6 & 7^e^ Slice 19 < Slices 3, 4, 6 & 7^e^
	Anterior / ML	6	2–9	12.76 (2.88)	p = 0.098^**a**^	-
	Middle / ML	15	2–12	15.83 (4.26)	**p = 0.028** ^**a**^	
	Posterior / ML	15	7–11	16.16 (4.93)	p = 0.073^a^	-
**Rabbit 3**	Anterior / ML	6	2–6	13.73 (2.37)	p = 0.447^a^	-
	Middle / ML	16	3–21	17.28 (4.01)	p = 0.437^a^	-
	Posterior / ML	11	3–10	18.80 (5.63)	p = 0.106^a^	-
	Anterior / VD	17	1–8	15.57 (3.61)	**p < 0.001** ^**a**^	Slice 16 < Slices 5 & 6^e^ Slice 17 < Slices 4-6^e^
	Middle / VD	19	6–16	17.67 (3.46)	**p < 0.001** ^**a**^	Slice 6 > Slices 18 & 19^e^ Slice 9 > Slice 18^e^ Slice 10 > Slices 13, 18 & 19^e^
	Posterior / VD	19	2–9	18.92 (5.20)	**p < 0.001** ^**a**^	Slice 1 < Slices 2–4, 6–9 & 11^c^ Slice 4 > Slices 15, 16 & 18^c^ Slice 6 > Slice 16^c^ Slice 17 < Slices 3, 4, 6–8 & 11^c^ Slice 19 < Slices 2–11 & 14^c^

Due to the shape of the skull, the bone volume present within each region varied, thus the total number of slices analysed per region was dependent on its location and orthogonal direction of measurement. For example, the middle region contained a range of bones including the frontal bones, maxilla and zygomatic arch, and bone was present throughout the majority of the ML depth (Fig 1A). Therefore, when analysing in the ML direction (Fig 1A), the middle region included more slices compared to the anterior region, as the former only contained the nasal region and thus covered less of the ML depth of the region (e.g., see number of slices for Rabbits 1 and 2, middle region vs anterior region, Table 1).

The middle region of Rabbit 1 was ground down by ~1mm in order to increase the number of slices (a total of 28 slices when slicing in the AP direction, Table 1), thus enabling the region to be analysed in two separate sub-regions (one anterior and one posterior to the midline of the region, Table 2). The middle region of Rabbit 3 was also analysed in two separate sub-regions; again, one anterior and one posterior to the midline of the region.

**Table 2:** The significance of variation in cortical bone elastic modulus within sub-regions. Sub-regions labelled “Part 1” are the anterior portion from the midline of the part; sub-regions labelled “Part 2” are from the posterior portion. Significance difference within a region was determined through an ^a^ANOVA (p < 0.05) or ^b^Kruskal-Wallis test. A ^c^Tukey’s post hoc or ^d^Dunn’s post hoc test determined the significant difference between slices within a region. Any slice with less than 2 indents was eliminated from the analysis. The direction in which a region was analysed is defined as: anteroposterior (AP), mediolateral (ML) and ventrodorsal (VD).

	Region / Slicing direction	No. of slices	No. of indents per slice	Average Elastic Modulus (GPa)	Significance between slices	
**Rabbit 1**	Middle Part 1 / AP	16	5–13	18.81 (3.52)	P = 0.266^a^	-
	Middle Part 2 / AP	12	4–10	16.46 (3.10)	p = 0.494^a^	-
**Rabbit 3**	Middle Part 1 / ML	14	2–11	17.77 (3.98)	p = 0.106^a^	-
	Middle Part 2/ ML	16	2–11	16.85 (4.02)	p = 0.601^a^	-
	Middle Part 1 / VD	18	1–8	18.11 (3.56)	**p = 0.003** ^**a**^	Slice 6 > Slices 17 & 18^c^
	Middle Part 2 / VD	19	3–11	17.38 (3.38)	**p < 0.001** ^**b**^	Slice 2 < Slice 10^d^

Several indentation measurements were taken on each slice in order to capture the range and variation of materials that were present: cortical and trabecular bone. Therefore, the number of indentations measured per slice was dependent on the area of the material(s) present within a single slice. Generally, there were more indentations per slice on cortical bone, when compared to trabecular bone (Tables 1 and 3). In total, the study involved the preparation and analysis of 18 bone sections and 2393 indentation measurements.

**Table 3:** The significance of variation in trabecular bone elastic modulus within a region. Statistical significance within a region was determined through either an ^a^Kruskal-Wallis (p < 0.05), ^b^Mann-Whitney U (p < 0.05) or ^c^ANOVA (p < 0.05) test. A ^d^Tukey’s post hoc test determined the significant difference between slices within a region. Any region with less than 2 slices, or any slice with less than 2 indents, was eliminated from the analysis. The direction in which a region was analysed is defined as: anteroposterior (AP), mediolateral (ML) and ventrodorsal (VD).

	Region / Slicing direction	No. of slices	No. of indents per slice	Average Elastic Modulus (GPa)	Significance between slices	
**Rabbit 1**	Anterior / AP	7	1–3	11.65 (4.75)	**p = 0.037** ^**a**^	
	Middle / AP	15	1–4	9.50 (2.49)	p = 0.382^a^	
	Posterior / AP	9	1–4	10.80 (3.51)	P = 0.064^a^	
	Anterior / ML	2	2–3	10.52 (1.88)	p = 0.564^b^	
	Middle / ML	10	1–4	8.41 (1.65)	p = 0.516^a^	
	Posterior / ML	7	1–3	9.60 (1.77)	p = 0.067^a^	
**Rabbit 2**	Anterior / AP	7	1–2	8.90 (3.46)	p = 0.439^b^	
	Middle / AP	10	1–3	10.29 (3.49)	p = 0.160^c^	
	Posterior / AP	18	1–6	10.31 (2.60)	p = 0.315^c^	
	Anterior / ML	1	2	7.11 (4.88)	-	
	Middle / ML	14	1–4	8.82 (2.83)	p = 0.150^c^	
	Posterior / ML	9	1–4	8.49 (1.70)	p = 0.316^c^	
**Rabbit 3**	Anterior / ML	3	1	8.33 (5.10)	-	
	Middle / ML	13	1–4	10.49 (3.58)	P = 0.322^a^	
	Posterior / ML	7	2–4	10.46 (3.30)	p = 0.927^c^	
	Anterior / VD	11	1–2	9.32 (3.09)	p = 0.952^c^	
	Middle / VD	17	1–8	11.51 (1.96)	p = 0.059^c^	
	Posterior / VD	17	1–4	10.79 (2.77)	**p = 0.001** ^**c**^	Slice 5> Slices 1, 2, 8 & 17^d^ Slice 11 > Slices 1, 2, 8 & 17^d^

Indentations were made with a load of 50 mN at (at 0.1 mN/nm), and held for 15 seconds (for the force–indentation depth curves created, see Supporting Information; S1-S12 Figs in S1 File). The elastic modulus of the material was determined using the Oliver-Pharr method [42] through initial calculation of the reduced modulus (Er) via Eq 1:

$$E_{r}=\frac{\sqrt{\pi }∙S}{2∙\beta ∙\sqrt{A_{p}\left(h_{c}\right)}}$$ (1)

Where β is the geometric factor of the Berkovich tip (since its ident shape is triangular, β = 1.034); A_p_ is the projected contact area and is calculated empirically based on indent depth h; h_c_ is the contact depth of the indenter with the sample at the set maximum test force F_max_ (in this setup, 50 mN); and, S is the contact stiffness, which is calculated as F_max_ divided by the distance between the maximum indentation depth and the tangent indentation depth.

The plane strain modulus (E*) was then calculated using Eq 2:

$$E^{*}=\frac{1}{\frac{1}{E_{r}}-\frac{1-{\vartheta_{i}}^{2}}{E_{i}}}$$ (2)

where E_i_ is the elastic modulus of the indenter (1141 GPa); and, ϑ_i_ is the Poisson’s ratio of the indenter (0.07). Finally, the indentation modulus (E_IT_) could be calculated via Eq 3:

$$E_{IT}=E^{*}∙\left(1-{\vartheta_{s}}^{2}\right)$$ (3)

where ϑ_s_ is the sample Poisson’s ratio (this was set to 0.3 for all materials).

One-way ANOVA and its non-parametric equivalent (Kruskal-Wallis), followed by post hoc tests (Tukey or Dunnett’s T3), were employed to assess differences in averaged cortical and trabecular bone elastic moduli: within each single region (i.e. testing for difference between individual slices); between different locations within the same individual (i.e. testing Hypothesis 1 through investigating difference between the anterior, middle and posterior regions); difference between individuals (i.e. testing Hypothesis 2); and, difference in each orthogonal direction (e.g., testing Hypothesis 3 through investigating difference in moduli measured AP, ML and VD). Data within each category (slice number, location of region, individual and orthogonal direction) were scrutinized to detect outliers, that were eventually removed before any statistical analysis.

## Results

### Variation of elastic modulus within a single region

**Fig 2:**
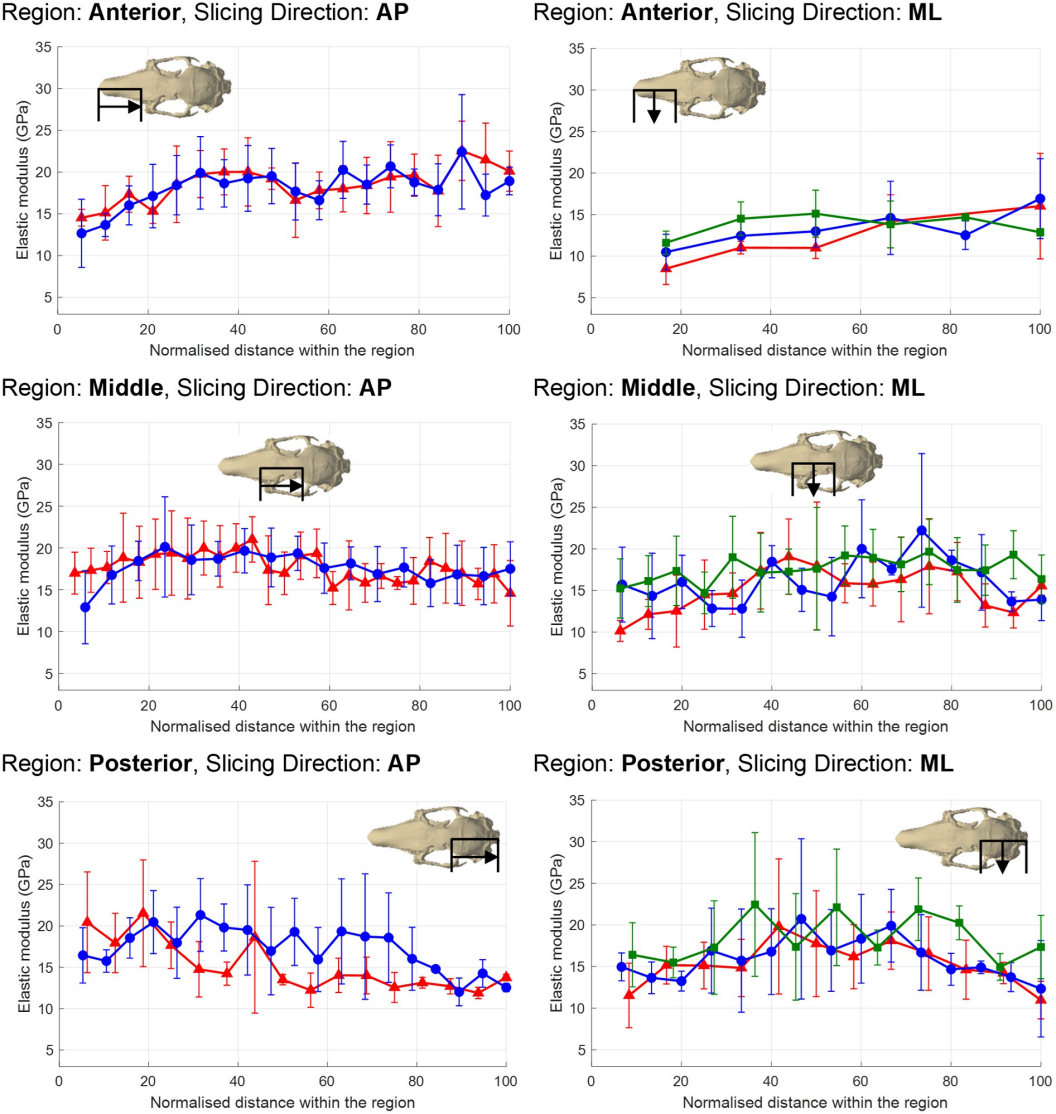
The average cortical bone elastic modulus (GPa) in each slice when measured in the anteroposterior (AP) and mediolateral (ML) directions. The position of a slice within a region is displayed as the normalised distance from either the anterior or medial surface of the region (i.e. 0% at the anterior/medial surface, 100% at the posterior/lateral surface). Error bars indicate ±1 standard deviation of the average. Red lines = data for Rabbit 1, blue lines = data for Rabbit 2, green lines = data for Rabbit 3.

**Fig 3:**
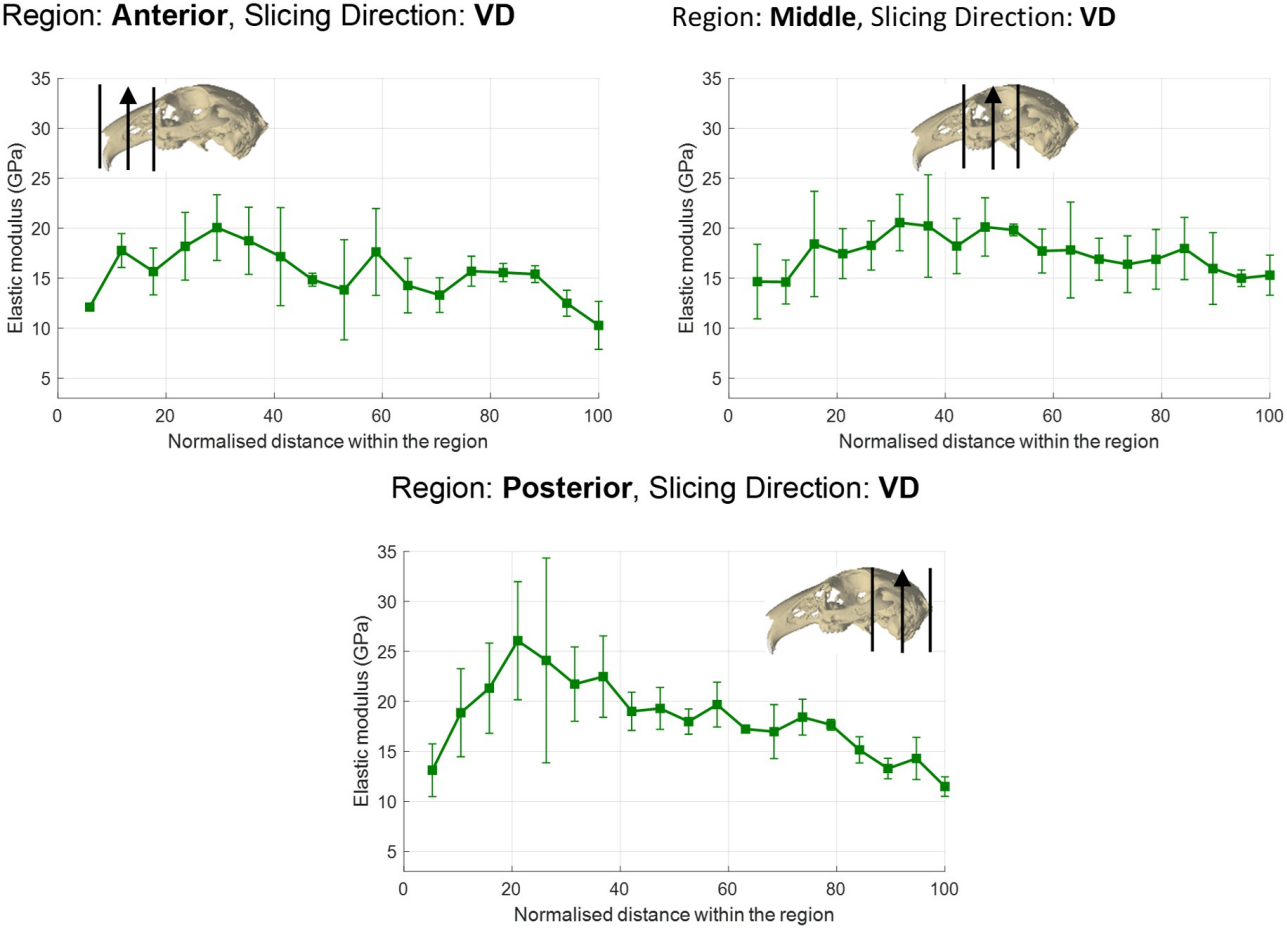
The average cortical bone elastic modulus (GPa) in each slice for Rabbit 3 when measured in the ventrodorsal (VD) direction. The position of slice within a region is displayed as the normalised distance from ventral surface of the region (i.e. 0% at the ventral surface, 100% at the dorsal surface). Error bars indicate ±1 standard deviation of the average.

The elastic modulus of cortical bone varied in all regions (anterior, middle and posterior), irrespective of individual or the orthogonal direction in which a region was measured (i.e. AP, ML or VD) (Figs 2 and 3) (for more details see Supporting Information; S13-S15 Figs in S1 File). The variation of cortical elastic modulus was most noticeable in: Rabbit 1 –a general decrease when moving through the posterior region when measured AP, and a general increase when moving through the anterior region when measured ML (Fig 2); Rabbit 2 –larger moduli when moving through the anterior region when measured ML (Fig 2); and, Rabbit 3 –larger moduli at the beginning of the anterior and posterior regions when measured VD (Fig 3).

The variation of cortical bone elastic modulus within a single region was not always significantly different between slices. For example, there was no significant difference between the moduli measured in individual slices in any region of Rabbit 3 when measured ML (Table 1). Likewise, there was no significant difference in Rabbit 2 between slices in the anterior (measured ML), middle (measured AP) and posterior (measured ML) regions. However, significant differences were observed in all other regions for each individual. Table 1 shows that such significant differences were generally attributed only to a difference between a small proportion of slices. For example, the posterior region (measured VD) of Rabbit 3 had the largest number of intra-slice significant differences, with 5 slices that differed from the rest; however, this only represented a proportion of the 19 slices analysed. Despite statistical significance between slices in the middle region (measured ML) of Rabbit 2, it was not possible to identify which slices caused the significant difference.

Analysis of sub-regions confirmed there was no statistical significance in the middle region of either Rabbits 1 and 3 (Table 2). The statistical significance in the middle region of Rabbit 3 (measured VD) (Table 1), was also observed in sub-regions.

Analysis of the elastic modulus in the trabecular bone found fewer instances of statistical significance between slices within a region, with no significant difference in any region of Rabbit 2 (Table 3). Significant differences were found in the anterior and posterior regions (both measured AP) of Rabbit 1 (although no statistical test relieved which slices were significantly different), and in the posterior region (measured VD) of Rabbit 3.

### Variation of elastic modulus between regions (same individual and orthogonal direction)

As the instances of significant difference were sporadic and limited to only a small volume within a region, any slice that was significantly different from another slice/s within the same region (Tables 1 and 3), was excluded from further analyses of the cortical and trabecular bone elastic modulus. In addition, when analysing the trabecular elastic modulus, any region which had less than 2 slices or only had slices with only one indent, were excluded from further analysis; this related to the anterior region (measured ML) of Rabbit 2, and the anterior region (measured ML) of Rabbit 3.

Analysis of the cortical bone elastic modulus found the following significant differences between regions, when measured in the same orthogonal direction: Rabbit 1—between the anterior and middle regions (both measured AP; 18.62 [SD = 3.60] GPa vs 17.94 [SD = 3.55] GPa), and between the anterior and posterior regions (both measured ML; 11.14 [SD = 3.32] GPa vs 15.78 [SD = 4.74] GPa); Rabbit 2—between the anterior and the other two regions (all measured ML; 12.76 [SD = 2.88] GPa vs 15.83 [SD = 4.26] GPa and 16.16 [SD = 4.93] GPa); and, Rabbit 3 –between the anterior and posterior regions (both measured ML; 13.73 [SD = 2.37] GPa vs 18.80 [SD = 5.63] GPa), and between the anterior and the other two regions (all measured VD; 15.57 [SD = 3.61] GPa vs 17.67 [SD = 3.46] GPa and 18.92 [SD = 5.20]) (Table 4).

**Table 4:** The significance of variation in cortical bone elastic modulus between regions in the skull. Significance difference between regions was determined through a Dunnett’s T3 post hoc test (p < 0.05). Regions are labelled as: Ant = anterior; Mid = middle; Post = posterior. The direction in which a region was analysed is defined as: anteroposterior (AP), mediolateral (ML) and ventrodorsal (VD).

**Rabbit 1**		Ant (AP)	Mid (AP)	Post (AP)	Ant (ML)	Mid (ML)	Post (ML)
	Ant (AP)	-	**0.003**	1.000	**0.000**	**0.000**	**0.000**
	Mid (AP)	-	-	0.538	**0.000**	**0.000**	**0.006**
	Post (AP)	-	-	-	**0.000**	**0.000**	**0.001**
	Ant (ML)	-	-	-	-	0.066	**0.003**
	Mid (ML)	-	-	-	-	-	0.725
**Rabbit 2**		Ant (AP)	Mid (AP)	Post (AP)	Ant (ML)	Mid (ML)	Post (ML)
	Ant (AP)	-	0.261	0.868	**0.000**	**0.000**	**0.000**
	Mid (AP)	-	-	1.000	**0.000**	**0.002**	**0.001**
	Post (AP)	-	-	-	**0.000**	**0.001**	**0.001**
	Ant (ML)	-	-	-	-	**0.002**	**0.001**
	Mid (ML)	-	-	-	-	-	1.000
**Rabbit 3**		Ant (ML)	Mid (ML)	Post (ML)	Ant (VD)	Mid (VD)	Post (VD)
	Ant (ML)	-	0.121	**0.000**	**0.005**	0.820	0.075
	Mid (ML)	-	-	0.099	**0.000**	0.953	0.990
	Post (ML)	-	-	-	**0.000**	**0.007**	0.999
	Ant (VD)	-	-	-	-	**0.000**	**0.000**
	Mid (VD)	-	-	-	-	-	0.634

Trabecular bone elastic modulus produced fewer instances of significant difference between regions, when tested in the same orthogonal direction. A significant difference was found between regions of Rabbit 1 (ANOVA, p = 0.027), although neither a Tukey’s or Dunnett’s T3 post hoc test could identify which individual regions were statistically different.

Significant difference was also present between all regions of Rabbit 2 (ANOVA, p = 0.006), although there was no difference between regions measured in the same orthogonal direction. No significant difference was found between any region of Rabbit 3 (ANOVA, p = 0.161).

### Variation of elastic modulus between individuals (same region and orthogonal direction)

Analysis of the cortical bone elastic modulus found significance differences between Rabbits 1 and 3 in the anterior and middle regions when measured ML (Table 5). This was due to the larger average elastic modulus in Rabbit 3 (at least 2.5 GPa larger in each region when compared to Rabbit 1), although no difference was found between Rabbits 1 and 2. Although significance was found between individuals in the posterior region (measured ML), it was not possible to determine which individual/s caused the difference. There was no significant difference between Rabbits 1 and 2 when measured AP.

**Table 5:** The significance of variation in cortical trabecular bone stiffness between specimens. Significance difference within a part was determined through either an ^a^Kruskal-Wallis (p < 0.05), ^b^ANOVA (p < 0.05) or ^c^Mann-Whitney U test (p < 0.05). The Homogeneity of Variance based on the median was used to determine an appropriate statistical method to test for significant differences between slices within a Part; either a ^d^Dunn’s post hoc test, ^e^Dunnett’s T3 post hoc test or ^f^Tukey’s post hoc test. The direction in which a part was analysed is defined as: anteroposterior (AP), mediolateral (ML) and ventrodorsal (VD). All specimens were tested in the ML direction. *Rabbits 1 & 2 were tested in the AP direction, and Rabbit 3 tested in the VD direction. ** no trabecular data for Rabbit 2 was present. ***no trabecular data for Rabbit 3 was present.

Region / Slicing direction	Cortical bone		Trabecular bone	
	Significance between specimens		Significance between specimens	
Anterior (ML)	**p < 0.001** ^**a**^	Rabbit 3 > Rabbits 1 & 2^d^	**p = 0.500^c^	
Middle (ML)	**p < 0.001** ^**b**^	Rabbit 3 > Rabbits 1 & 2^e^	**p = 0.006** ^**b**^	Rabbit 3 > Rabbits 1 & 2^e^
Posterior (ML)	**p < 0.001** ^**a**^		**p = 0.008** ^**b**^	Rabbit 2 < Rabbit 3^f^
Anterior (AP or VD)*	**P < 0.001** ^**b**^	Rabbit 3 < Rabbits 1 & 2^f^	***p = 0.886^c^	-
Middle (AP or VD)*	p = 0.162^b^	-	p = 0.231^b^	-
Posterior (AP or VD)*	p = 0.480^a^	-	p = 0.604^b^	-

No statistically significant differences were found for the trabecular elastic modulus between individuals in any region measured AP (Table 5).

### Variation of elastic modulus when measured in different orthogonal directions (same individual and region)

Analysis of the cortical bone elastic moduli measured in different orthogonal directions, within a single region (i.e. anterior region measured AP vs anterior region measured ML) (Table 4), found several instances of statistically significant differences. For example, all regions of Rabbits 1 and 2 contained significant differences between moduli measured in AP and ML. Rabbit 3 displayed less significant differences, with only the anterior region differing between ML and VD.

Less variation was observed in the trabecular bone elastic modulus, with significant differences present only in Rabbit 2, with a Tukey’s post hoc test determining a difference in the middle region between moduli measured in AP and ML (p = 0.041). While a significant difference was found between all regions in Rabbit 1, it was not possible to determine if this was due to moduli measured in different orthogonal directions, while there was no significance difference in any region of Rabbit 3.

## Discussion

This study has, for the first time, measured the regional variation and orthotropic nature of the cortical and trabecular bone elastic modulus in the rabbit skull. Statistical tests have shown that the elastic modulus not only varies between regions, but also with orthogonal direction. The elastic modulus of cortical bone was observed to vary between slices within all regions (Figs 2 and 3). There were no clear patterns to characterise these variations in terms of the anatomical location, the specimen or the direction of the measurement. The range of cortical moduli within a single region varied, with some regions containing moduli within a 5 GPa range (e.g., the middle region of Rabbit 1 when measured AP, anterior region of Rabbit 2 when measured ML, or middle region of Rabbit 3 when measured VD), while in others the range exceeded 10 GPa (e.g., anterior region of Rabbit 2 when measured AP, and posterior region of Rabbit 3 when measured VD). These variations were statistically significant in the majority of regions (Table 1). Rabbit 3 was the only specimen with no significant difference in any regions when measured in the same direction (AP direction), whereas significance was found in all regions of Rabbit 1, irrespective of direction of measurement. However, significant difference within a single region was generally attributed to a maximum of 5 slices (e.g., posterior region of Rabbit 3 when measured VD), which was only a proportion (maximum of 27%) of the total number of slices analysed in each region. Therefore, as these slices represented only a small volume within a region, it was deemed reasonable to eliminated the slice/s causing the significant difference in order to test Hypotheses 1 and 3.

The presence of statistical significance between slices within a region was not necessarily sensitive to the actual size of the region (Fig 1). For example, when the middle region of Rabbit 3 (measured ML) was analysed in two separate sub-regions, both sub-regions contained statistically different parts (Table 2), as did the analysis of the whole region (Table 1). The middle region of Rabbit 1 (when measured AP) was an exception, as the separate sub-regions contain no significant difference, proving that the difference observed within the whole region (Table 1) was caused by two slices positioned on either side of the region’s midline. This confirmed that the difference was limited only to a small volume of the region.

We are not aware of any previous attempts to measure the elastic modulus of bone in the rabbit skull, therefore direct comparison of the values in the study is not possible. A variation of elastic modulus values are often found within literature due to the range of experimental methods used, however the average cortical elastic modulus values in this study (ranging between 11.14–18.92 GPa; Table 1) are within the upper limits of those measured in the human skull [23, 25, 43, 44]. Furthermore, the average cortical elastic modulus values in this study are also within the range of values used in FEA modelling of the skull in a range of taxa [10, 11, 13, 15, 19, 29–31]. The elastic modulus of trabecular bone in each region was generally lower (ranging between 7.11–11.65 GPa) than that of cortical bone (cf. Tables 1 and 3). The elastic modulus was only similar between the two types of bone in one instance (anterior region of Rabbit 1 when measured ML), otherwise, the modulus of cortical bone was ~1.5–2 times larger in magnitude. This finding is similar to other studies which have reported a larger cortical elastic modulus [4–6]. There were fewer instances of statistical significance between slices within a region when analysing the trabecular bone (Table 2). Observations of trabecular bone in this study are made with respect to lower number of indents per slice, and slices per region, when compared to cortical bone. Thus statistical calculations are based on a smaller dataset, however, this is unavoidable due to the smaller volume present for trabecular bone, and difficulties in accurately differentiating it from cortical bone.

Comparison between regions identified that both the cortical and trabecular bone elastic moduli increase when moving posteriorly through the skull, when measured ML (with the exception of trabecular bone in Rabbit 1) and VD. In comparison, the elastic modulus was more evenly distributed when measured AP (with the exception of cortical bone in Rabbit 1). Significant differences were observed between at least two regions in all individuals when measured in the same direction, with the exception of Rabbit 2 when measured AP (Table 4). These differences were always present between the anterior and posterior regions when measured in both ML and VD, although interestingly there was no significant difference between the middle and posterior region in any orthogonal direction. The latter may be considered surprising considering the zygomatic arch and maxilla are predicted to experience higher masticatory strains than those of the parietal and occipital bones in the rabbit [31]. However, it is important to note that the posterior region contained the volume around the temporomandibular joint, which also experiences high strains during mastication [31]. No significant difference was found in the trabecular bone between regions measured in the same orthogonal direction in Rabbits 2 and 3, while it was not possible to determine which region(s) or orthogonal direction caused a significant difference in Rabbit 1. Therefore, evaluation of Hypothesis 1 is dependent on the type of bone: it is rejected for cortical bone for all orthogonal directions; and, it is accepted for trabecular bone.

The statistical significance of both cortical and trabecular elastic modulus between specimens was found to be dependent on the direction of measurement. Rabbit 3 was often different from all other individuals when measured ML (Table 5), however there was no difference between Rabbit 1 and Rabbit 2 in either AP or ML. Since not all specimens were measured AP, Rabbits 1 and 2 were subsequently compared to Rabbit 3 when the latter was measured VD. However, despite comparing individuals tested in different orthogonal directions, Rabbit 3 was only significantly different from the others in the anterior region when measuring cortical bone (Table 5). Consequently, evaluation of Hypothesis 2 is dependent on the orthogonal direction of measurement: it is rejected for elastic moduli measured in ML; and, it is accepted for elastic moduli measured in AP. Since only one specimen was measured VD, it is not possible to evaluate the hypothesis for this orthogonal direction.

When comparing the cortical and trabecular elastic modulus in a region when measured in different orthogonal directions, moduli measured ML were lower than that measured in the other two directions; for example, the average cortical elastic modulus in Rabbit 1 ranged between 11.14–15.78 GPa when measured ML, compared to 16.32–18.62 GPa when measured AP. Orthotropic properties were statistically significant in at least one region for all specimens, for both cortical and trabecular bone, with the only exception of trabecular bone in Rabbit 3. Therefore, Hypothesis 3 is rejected, with some specimens displaying significantly different orthotropic properties in all regions of the skull (e.g., Rabbits 1 and 2 for cortical bone), while in others it was localised to just one region.

The above observations are made with respect to the limitations of the methodology. Firstly, the distinction between cortical and trabecular bone was subjective and determined by the operator through visual inspection of each slice. To reduce potential error, indents were measured at locations away from the cortical–trabecular boundary, and no idents were measured in areas where it was difficult to determine a distinction between cortical and trabecular bone. Secondly, the nano-indentation procedure used in the methodology has been suggested to overestimate the hardness, sometimes by as much as 50% [45, 46], while the shape of the indenter is known to influence measured material properties [47]. Therefore, we used a Berkovich tip due to its use in previous studies of bone material properties [21, 40, 41], while comparisons with modulus values reported in literature confirmed the values reported here are unlikely to be significantly overestimated.

The finding that the heterogeneity between regions, and orthotropic properties, are often statistically significant, suggests that the choice of material modelling in FEA modelling of the rabbit skull (and eventually that of many more other mammals yet to be tested) should carefully consider the choice of material properties, especially when using homogenous isotropic elastic modulus. In conclusion, while it is recommended that future FEA studies the skull should aim to represent the heterogeneous, orthotropic nature of the bone, this is challenging and often impractical to implement. Therefore, it is recommended that homogenous, isotropic studies of the rabbit skull (and possibly other lagomorphs) should use elastic modulus values measured from cortical bone, and avoid using measurements taken from the anterior region of the skull (since it often differs significantly from the other regions) or those measured in the ML direction (since it is lower than the other directions). The dataset presented in this study will enable further investigation as to the influence of modelling bone properties with varying complexity (e.g. homogenous, isotropic vs heterogeneous, orthotropic) on biomechanical modelling of important applications, such as the use of rabbit models in the 3Rs of animal experiments.

## Supporting information

S1 File (PDF)
