# Supplementary material for: Regional variation of the cortical and trabecular bone material properties in the rabbit skull
Source: PLoS One. 2024 Feb 27;19(2):e0298621. doi: 10.1371/journal.pone.0298621 (PMC10898762; doi:10.1371/journal.pone.0298621)
Supplement: S1 File — (PDF) [file pone.0298621.s001.pdf]

Region: **Anterior**, Slicing Direction: **AP**

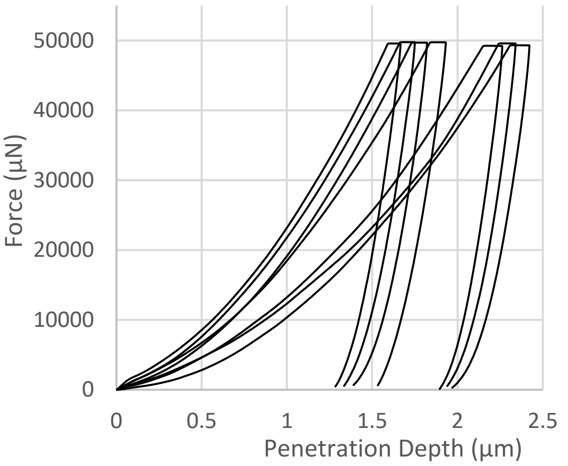


Region: **Anterior**, Slicing Direction: **ML**

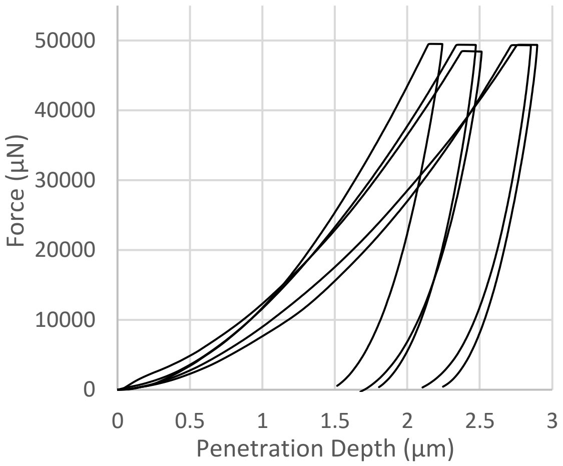


Region: **Middle**, Slicing Direction: **AP**

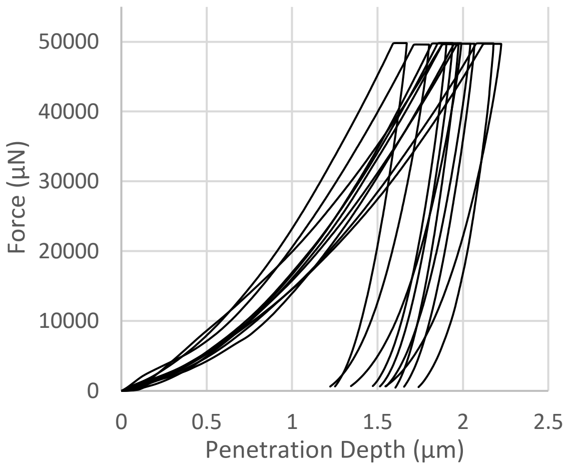


Region: **Middle**, Slicing Direction: **ML**

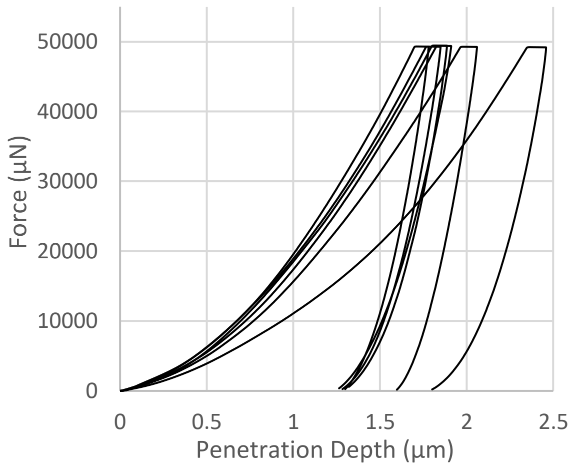


Region: **Posterior**, Slicing Direction: **AP**

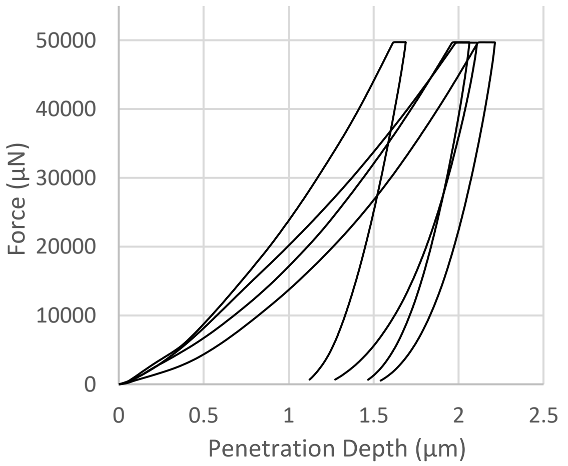


Region: **Posterior**, Slicing Direction: **ML**

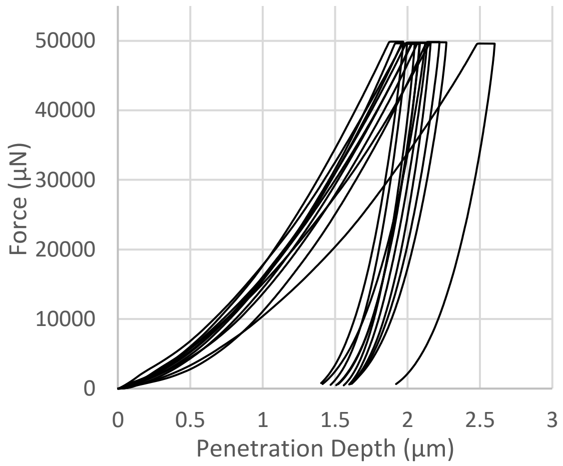


Figure S1. Sample force - penetration depth curves for indentations on the cortical bone of Rabbit 1. The direction in which a region was analysed is defined as: anteroposterior (AP) and mediolateral (ML).

Region: **Anterior**, Slicing Direction: **AP**

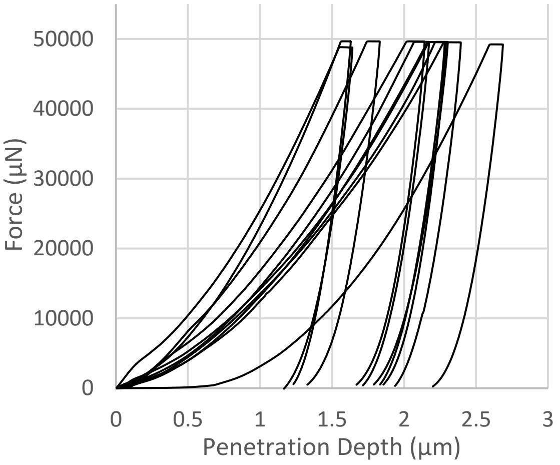


Region: **Anterior**, Slicing Direction: **ML**

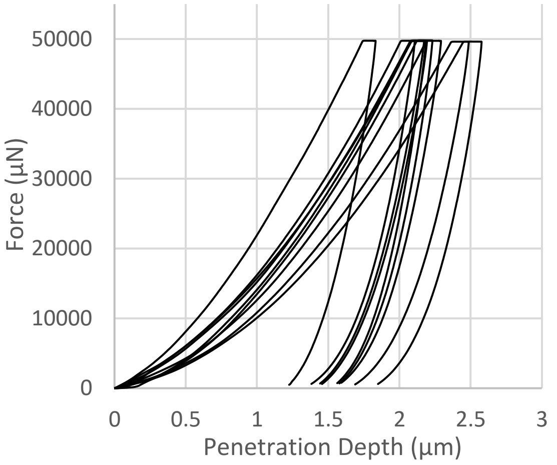


Region: **Middle**, Slicing Direction: **AP**

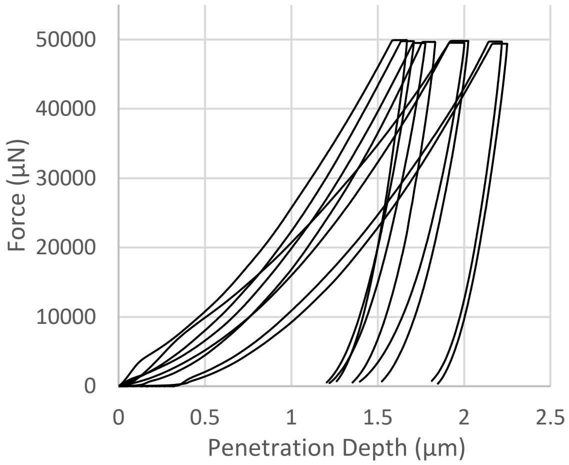


Region: **Middle**, Slicing Direction: **ML**

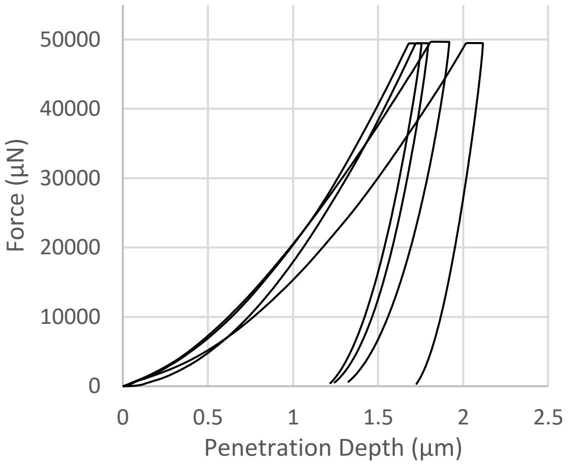


Region: **Posterior**, Slicing Direction: **AP**

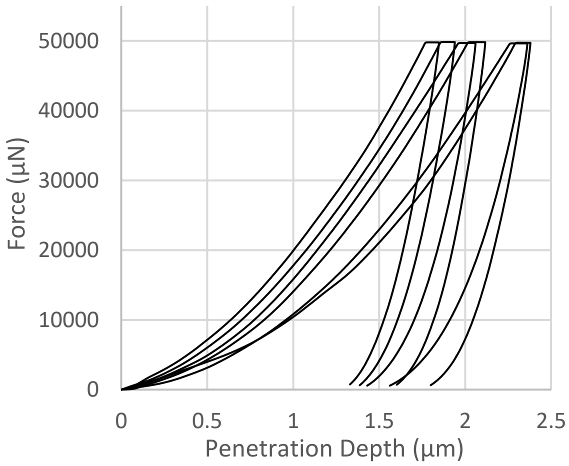


Region: **Posterior**, Slicing Direction: **ML**

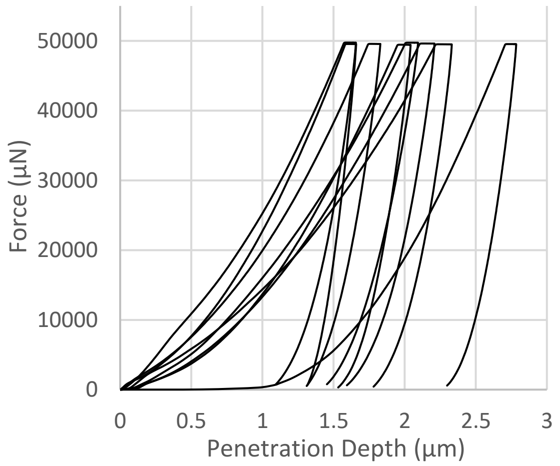


Figure S2. Sample force - penetration depth curves for indentations on the cortical bone of Rabbit 2. The direction in which a region was analysed is defined as: anteroposterior (AP) and mediolateral (ML).

Region: **Anterior**, Slicing Direction: **VD**

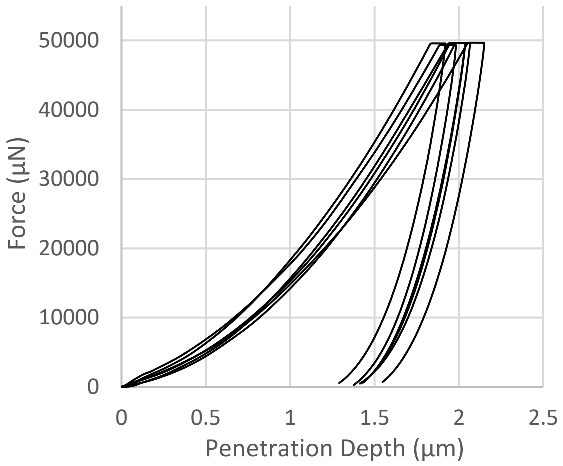


Region: **Anterior**, Slicing Direction: **ML**

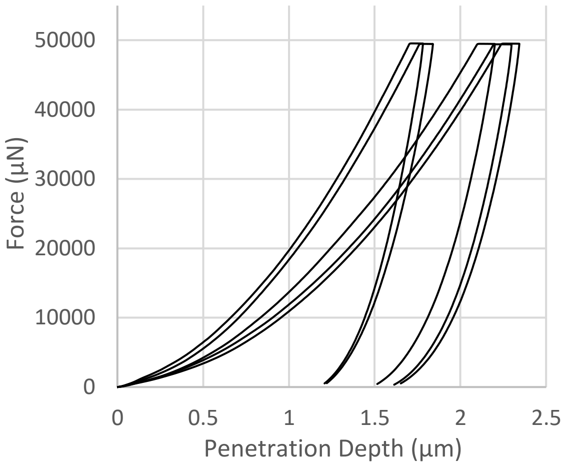


Region: **Middle**, Slicing Direction: **VD**

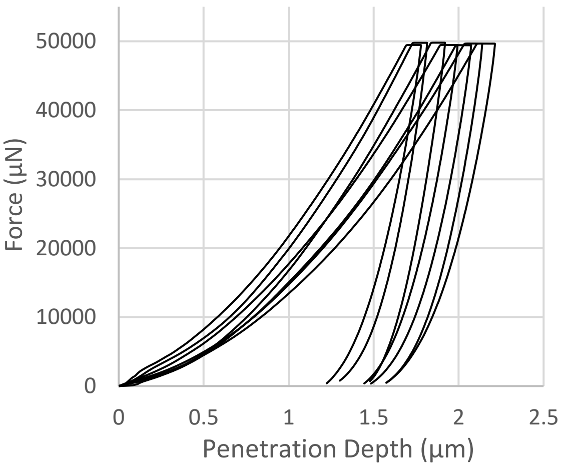


Region: **Middle**, Slicing Direction: **ML**

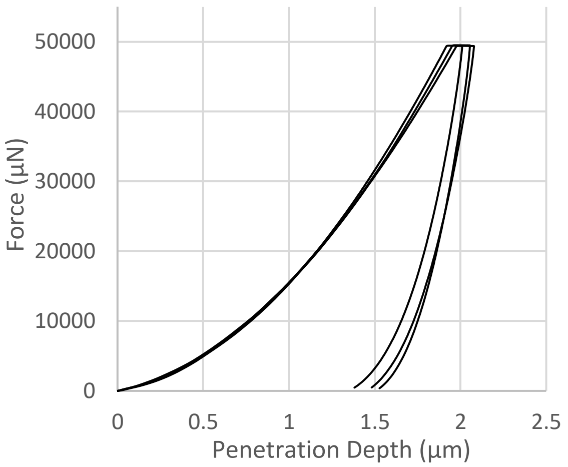


Region: **Posterior**, Slicing Direction: **VD**

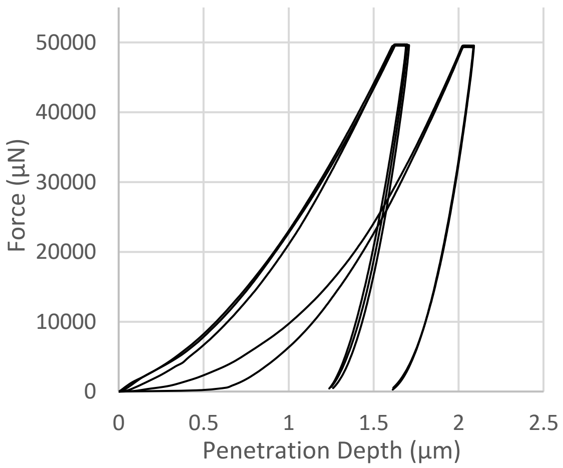


Region: **Posterior**, Slicing Direction: **ML**

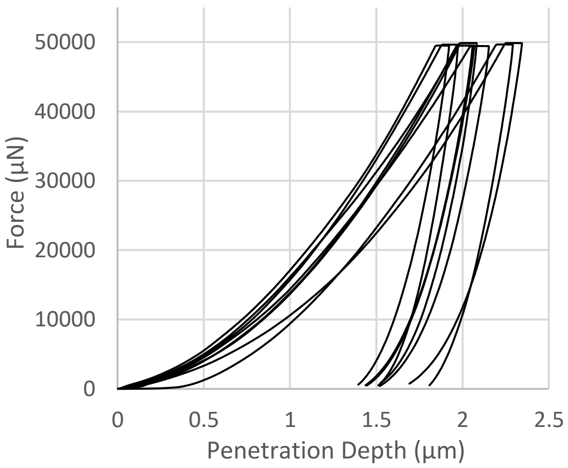


Figure S3. Sample force - penetration depth curves for indentations on the cortical bone of Rabbit 3. The direction in which a region was analysed is defined as: ventrodorsal (VD) and mediolateral (ML).

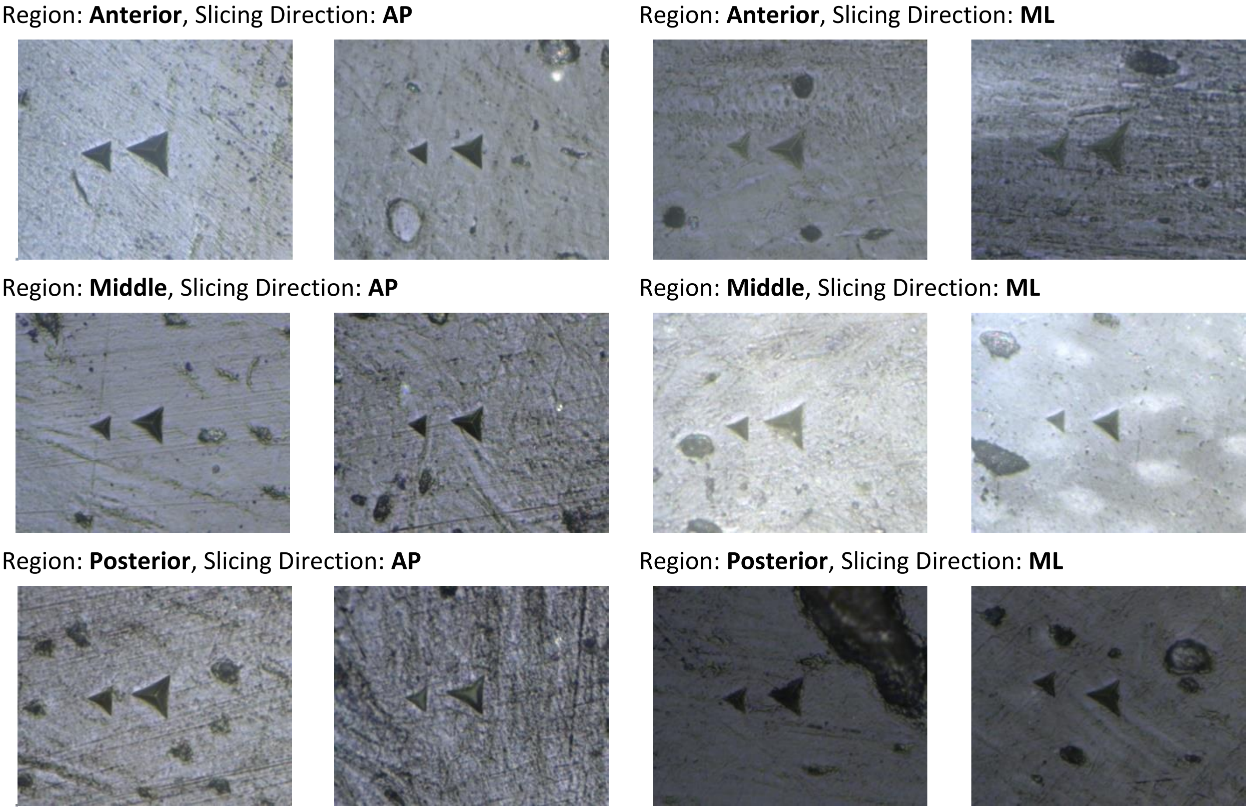


Figure S4. Idents made on the cortical bone (x50 magnification) of Rabbit 1. The direction in which a region was analysed is defined as: anteroposterior (AP) and mediolateral (ML).

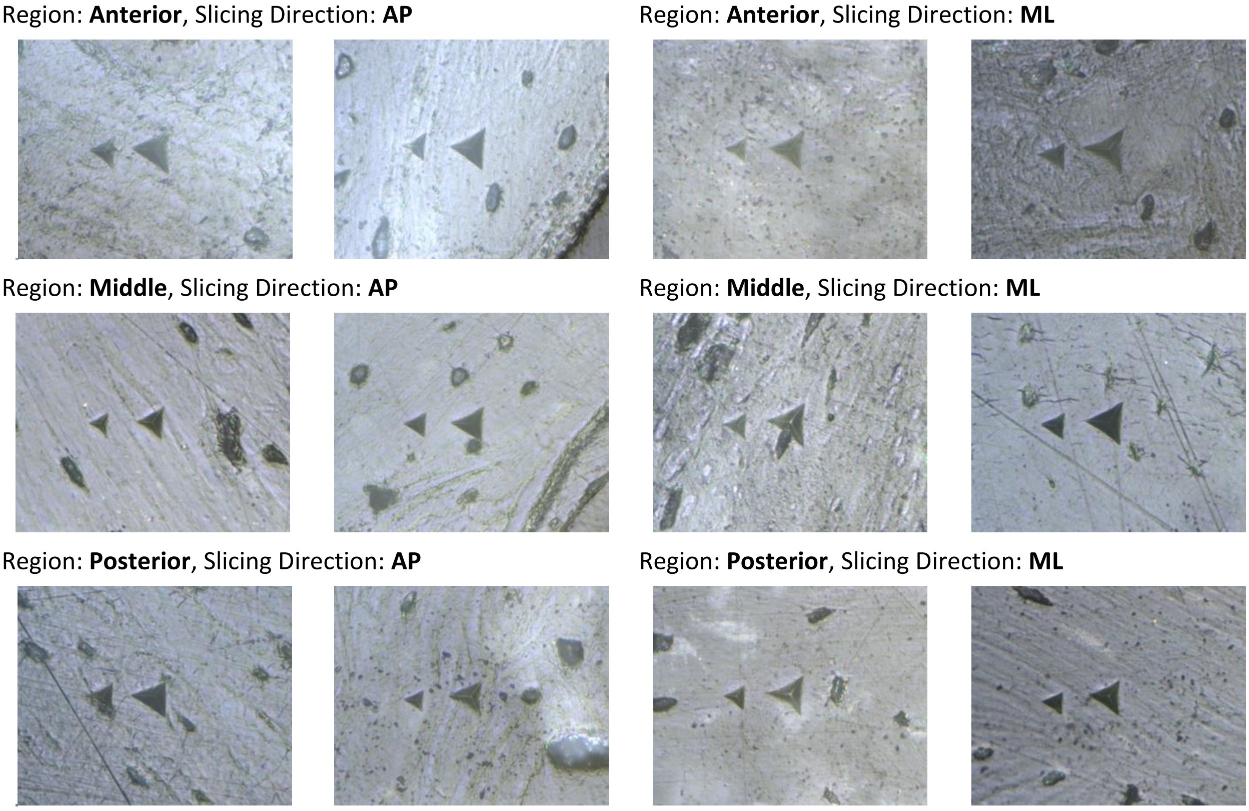


Figure S5. Idents made on the cortical bone (x50 magnification) of Rabbit 2. The direction in which a region was analysed is defined as: anteroposterior (AP) and mediolateral (ML).

Region: **Anterior**, Slicing Direction: **VD**

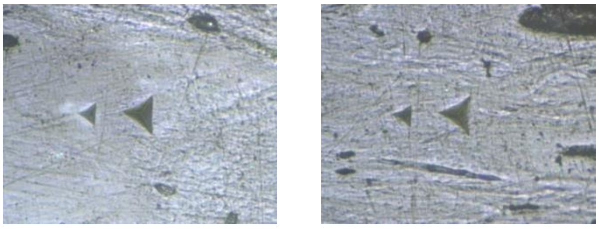

Region: **Anterior**, Slicing Direction: **ML**

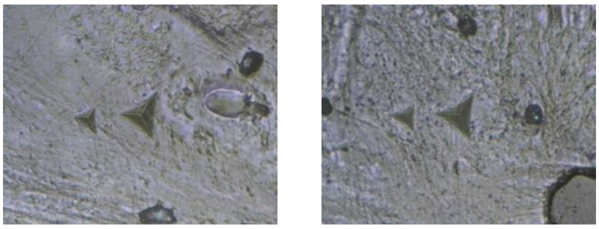

Region: **Middle**, Slicing Direction: **VD**

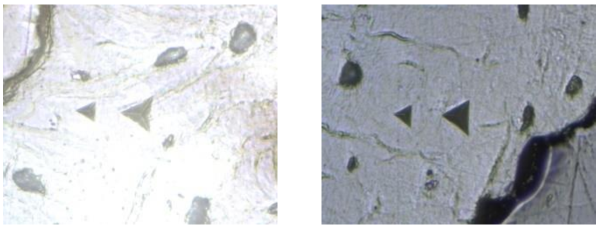

Region: **Middle**, Slicing Direction: **ML**

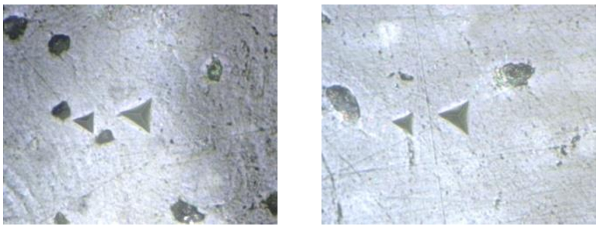

Region: **Posterior**, Slicing Direction: **VD**

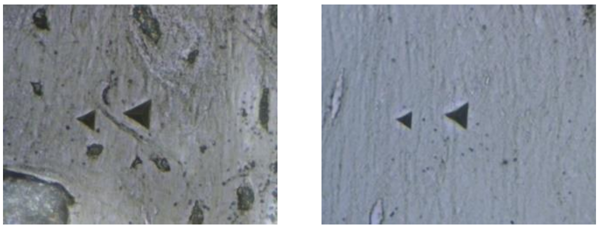

Region: **Posterior**, Slicing Direction: **ML**

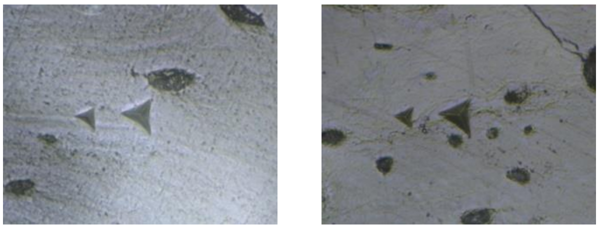

Figure S6. Idents made on the cortical bone (x50 magnification) of Rabbit 3. The direction in which a region was analysed is defined as: ventrodorsal (VD) and mediolateral (ML).

Region: **Anterior**, Slicing Direction: **AP**

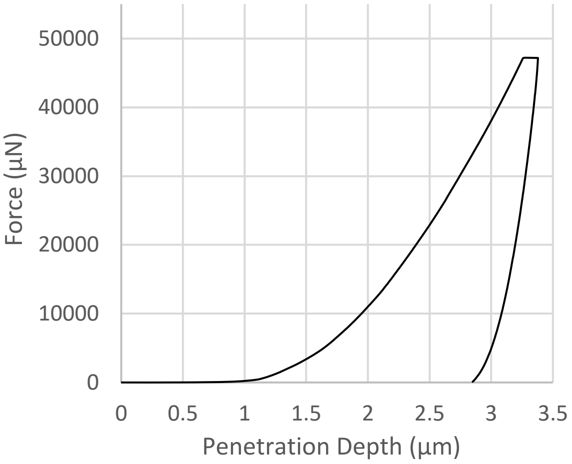


Region: **Anterior**, Slicing Direction: **ML**

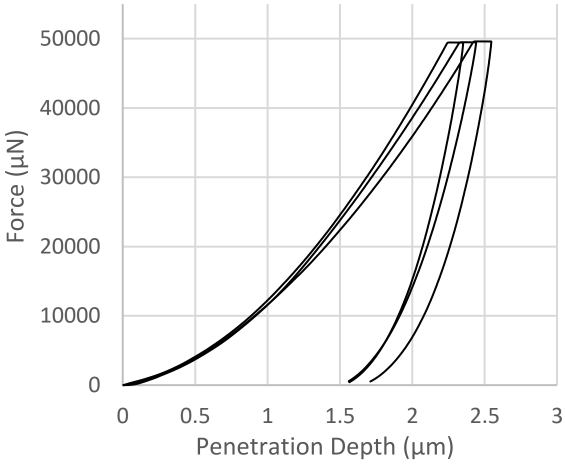


Region: **Middle**, Slicing Direction: **AP**

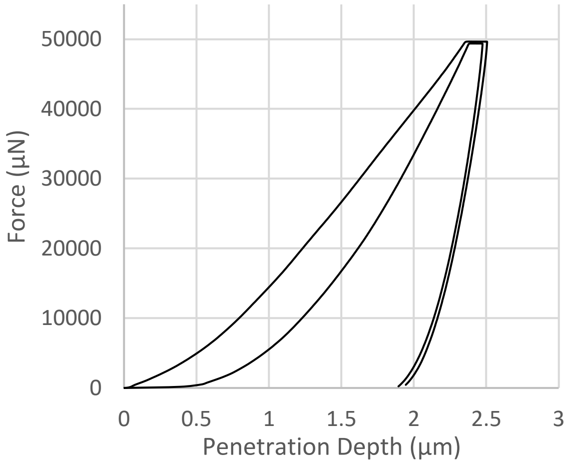


Region: **Middle**, Slicing Direction: **ML**

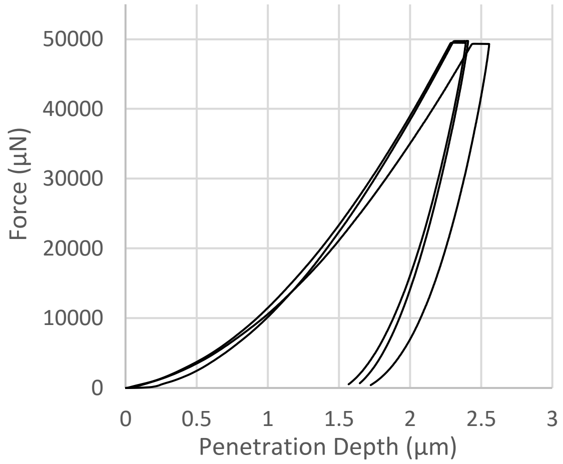


Region: **Posterior**, Slicing Direction: **AP**

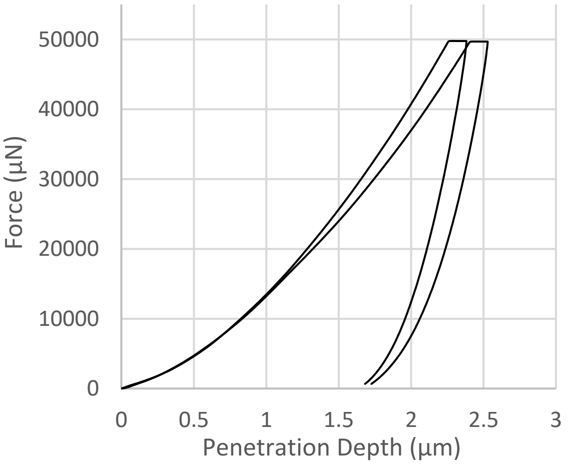


Region: **Posterior**, Slicing Direction: **ML**

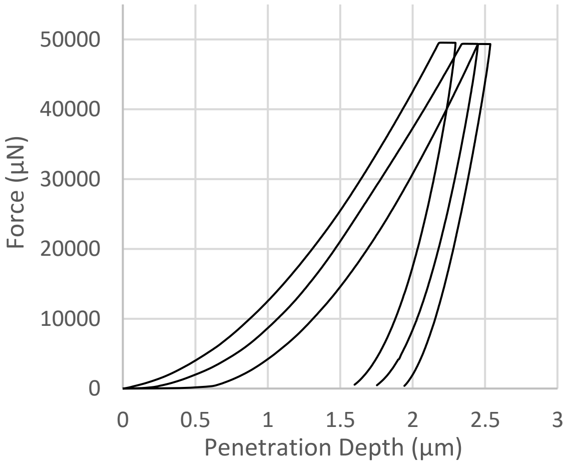


Figure S7. Sample force - penetration depth curves for indentations on the trabecular bone of Rabbit 1. The direction in which a region was analysed is defined as: anteroposterior (AP) and mediolateral (ML).

Region: **Anterior**, Slicing Direction: **AP**

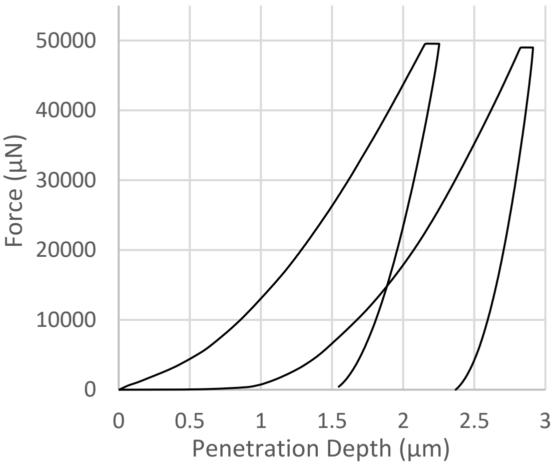


Region: **Anterior**, Slicing Direction: **ML**

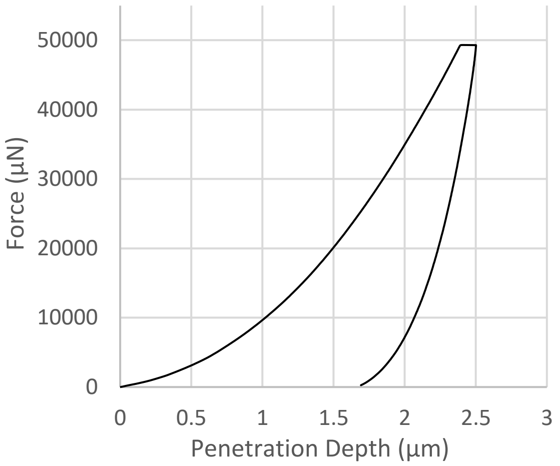


Region: **Middle**, Slicing Direction: **AP**

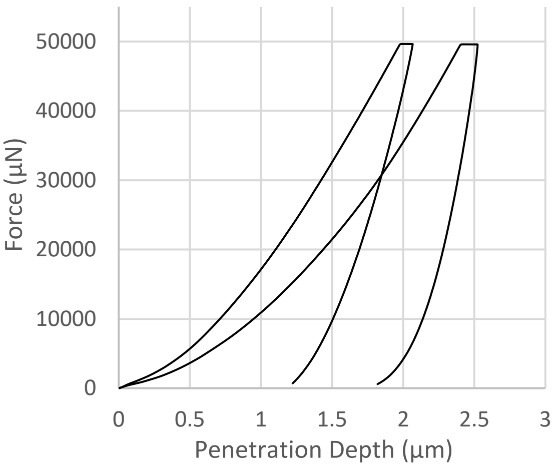


Region: **Middle**, Slicing Direction: **ML**

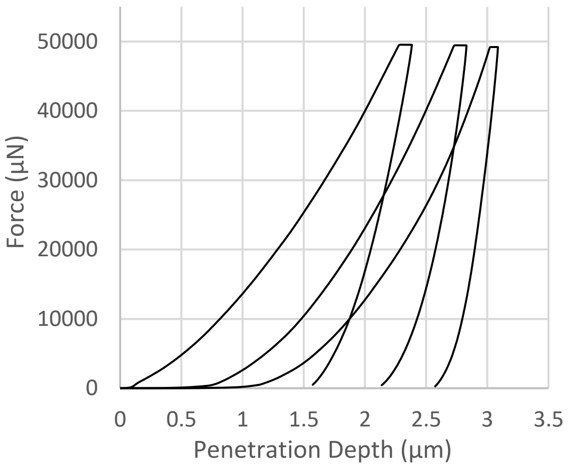


Region: **Posterior**, Slicing Direction: **AP**

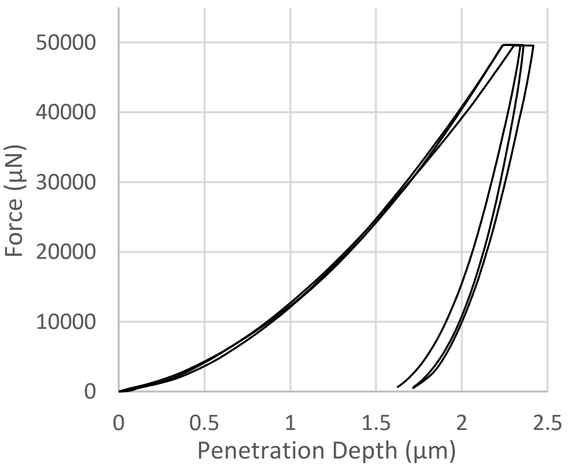


Region: **Posterior**, Slicing Direction: **ML**

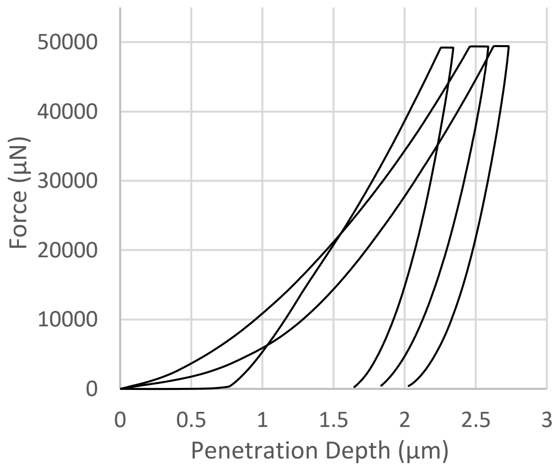


Figure S8. Sample force - penetration depth curves for indentations on the trabecular bone of Rabbit 2. The direction in which a region was analysed is defined as: anteroposterior (AP) and mediolateral (ML).

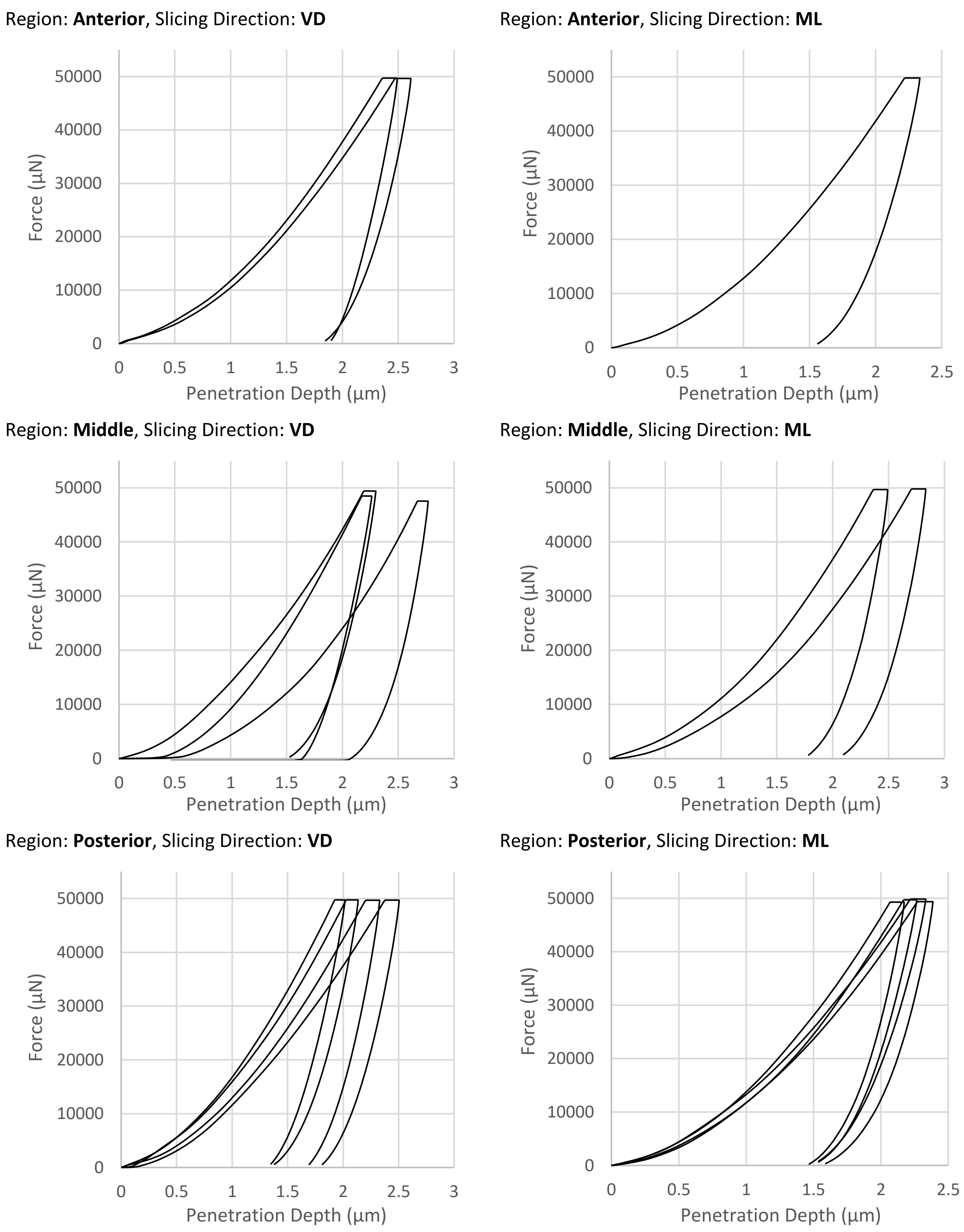


Figure S9. Sample force - penetration depth curves for indentations on the trabecular bone of Rabbit 3. The direction in which a region was analysed is defined as: ventrodorsal (VD) and mediolateral (ML).

Region: **Anterior**, Slicing Direction: **AP** Region: **Anterior**, Slicing Direction: **ML**

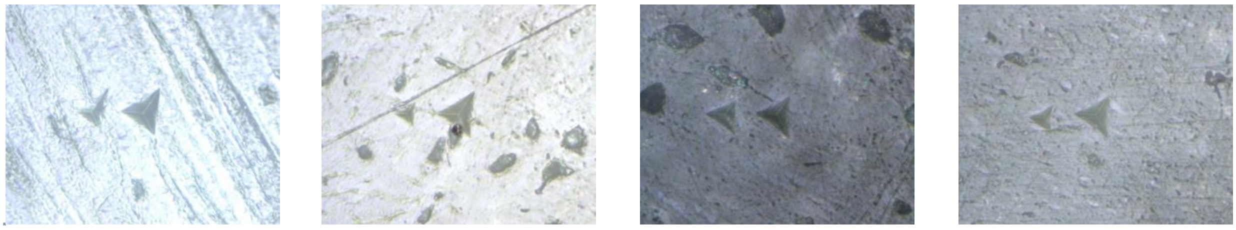

Region: **Middle**, Slicing Direction: **AP** Region: **Middle**, Slicing Direction: **ML**

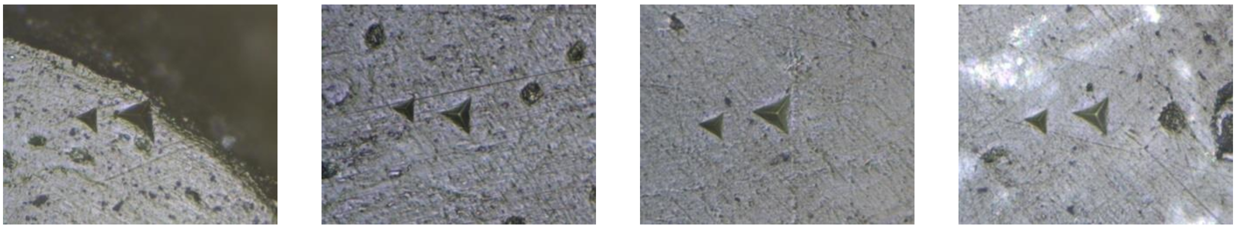

Region: **Posterior**, Slicing Direction: **AP** Region: **Posterior**, Slicing Direction: **ML**

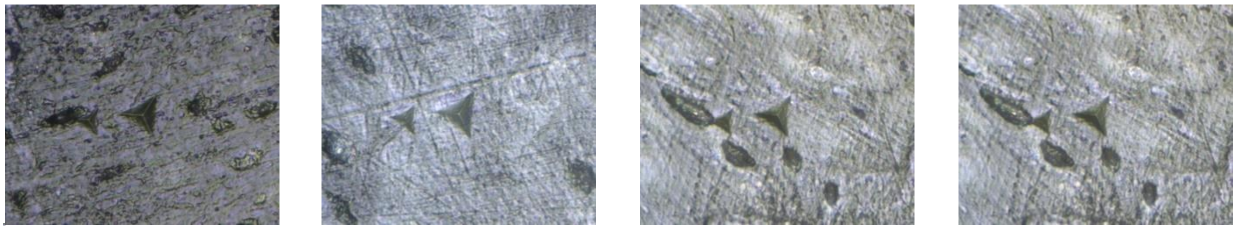

Figure S10. Idents made on the trabecular bone (x50 magnification) of Rabbit 1. The direction in which a region was analysed is defined as: anteroposterior (AP) and mediolateral (ML).

Region: **Anterior**, Slicing Direction: **AP** Region: **Anterior**, Slicing Direction: **ML**

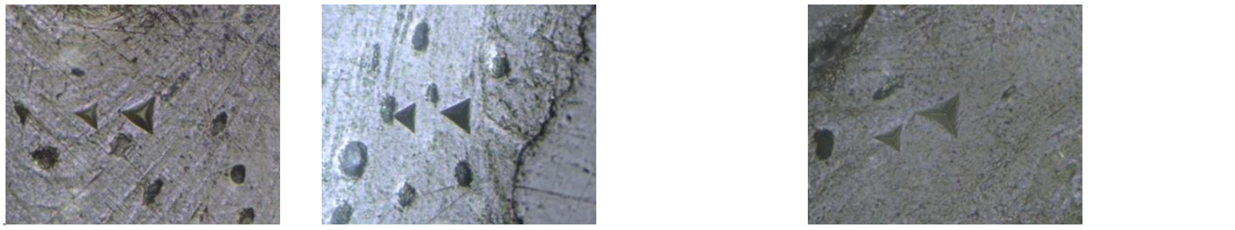

Region: **Middle**, Slicing Direction: **AP** Region: **Middle**, Slicing Direction: **ML**

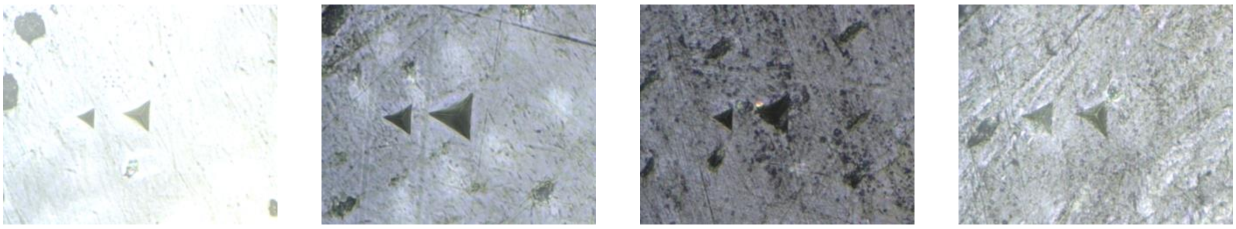

Region: **Posterior**, Slicing Direction: **AP** Region: **Posterior**, Slicing Direction: **ML**

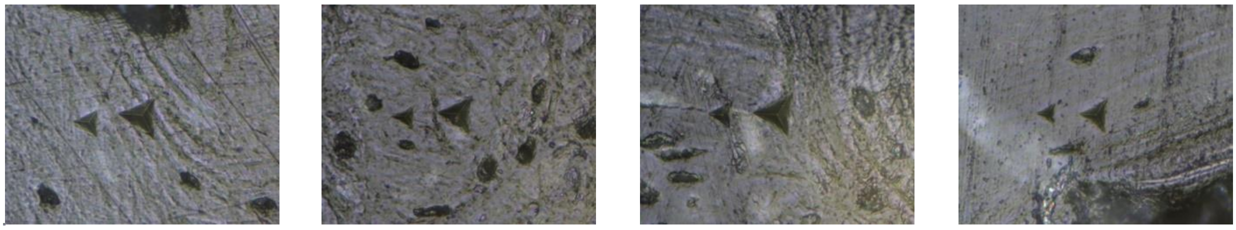

Figure S11. Idents made on the trabecular bone (x50 magnification) of Rabbit 2. The direction in which a region was analysed is defined as: anteroposterior (AP) and mediolateral (ML).

Region: **Anterior**, Slicing Direction: **ML**

Region: **Anterior**, Slicing Direction: **VD**

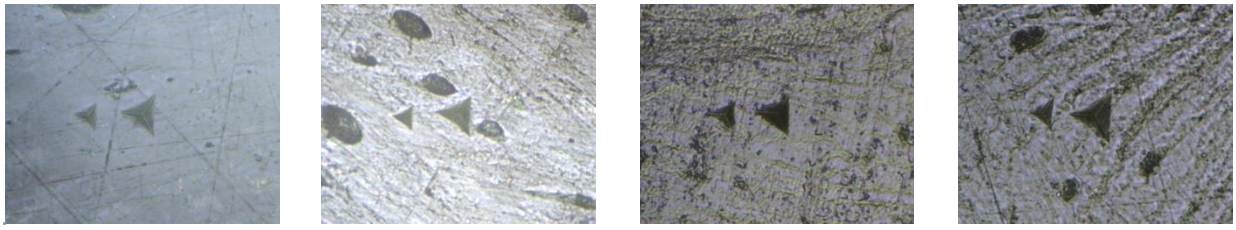

Region: **Middle**, Slicing Direction: **ML**

Region: **Middle**, Slicing Direction: **VD**

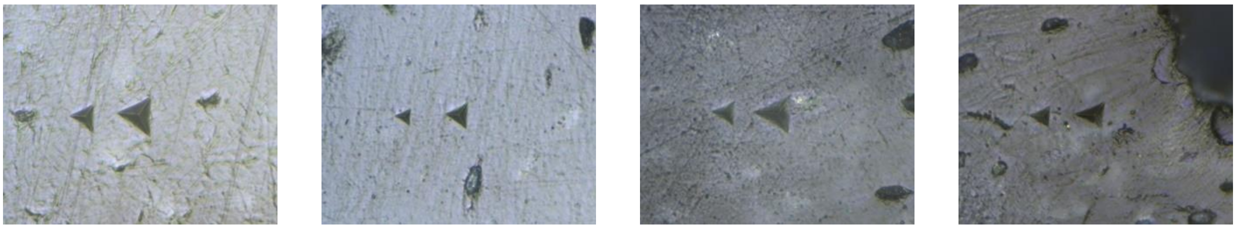

Region: **Posterior**, Slicing Direction: **ML**

Region: **Posterior**, Slicing Direction: **VD**

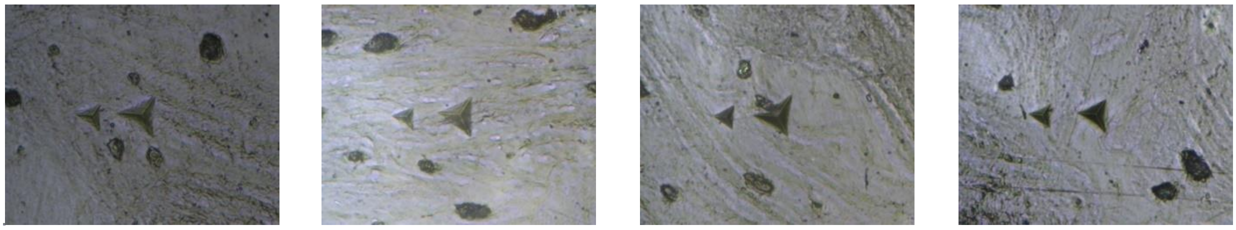

Figure S12. Idents made on the trabecular bone (x50 magnification) of Rabbit 3. The direction in which a region was analysed is defined as: anteroposterior (AP) and mediolateral (ML).

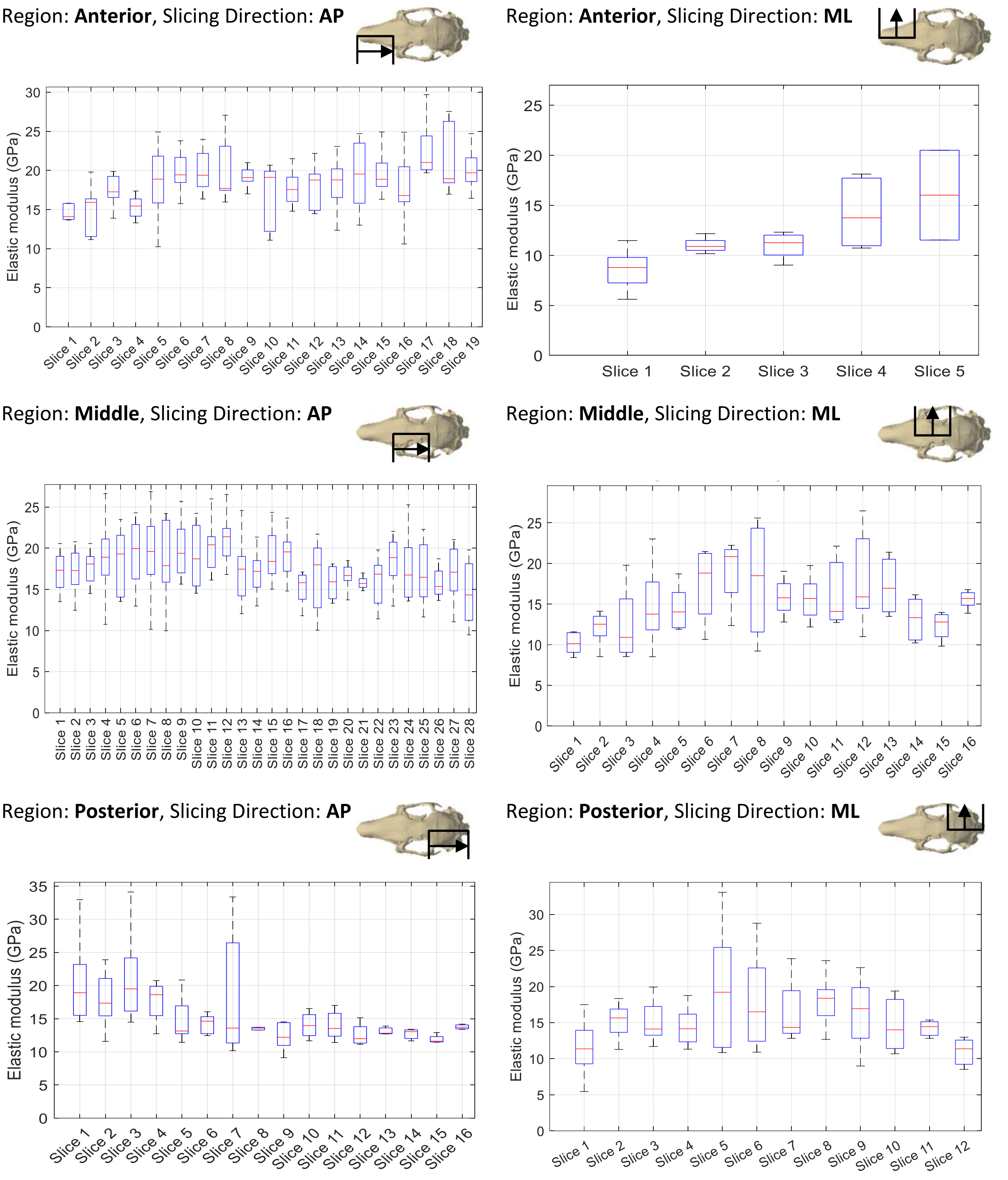


Figure S13. A box plot showing the minimum, first quartile, median, third quartile, and maximum elastic modulus measured in each slice of a region in Rabbit 1. The direction in which a region was analysed is defined as: anteroposterior (AP) and mediolateral (ML). Images accompanying each box plot illustrate the location of each region in the skull and the direction of the slicing.

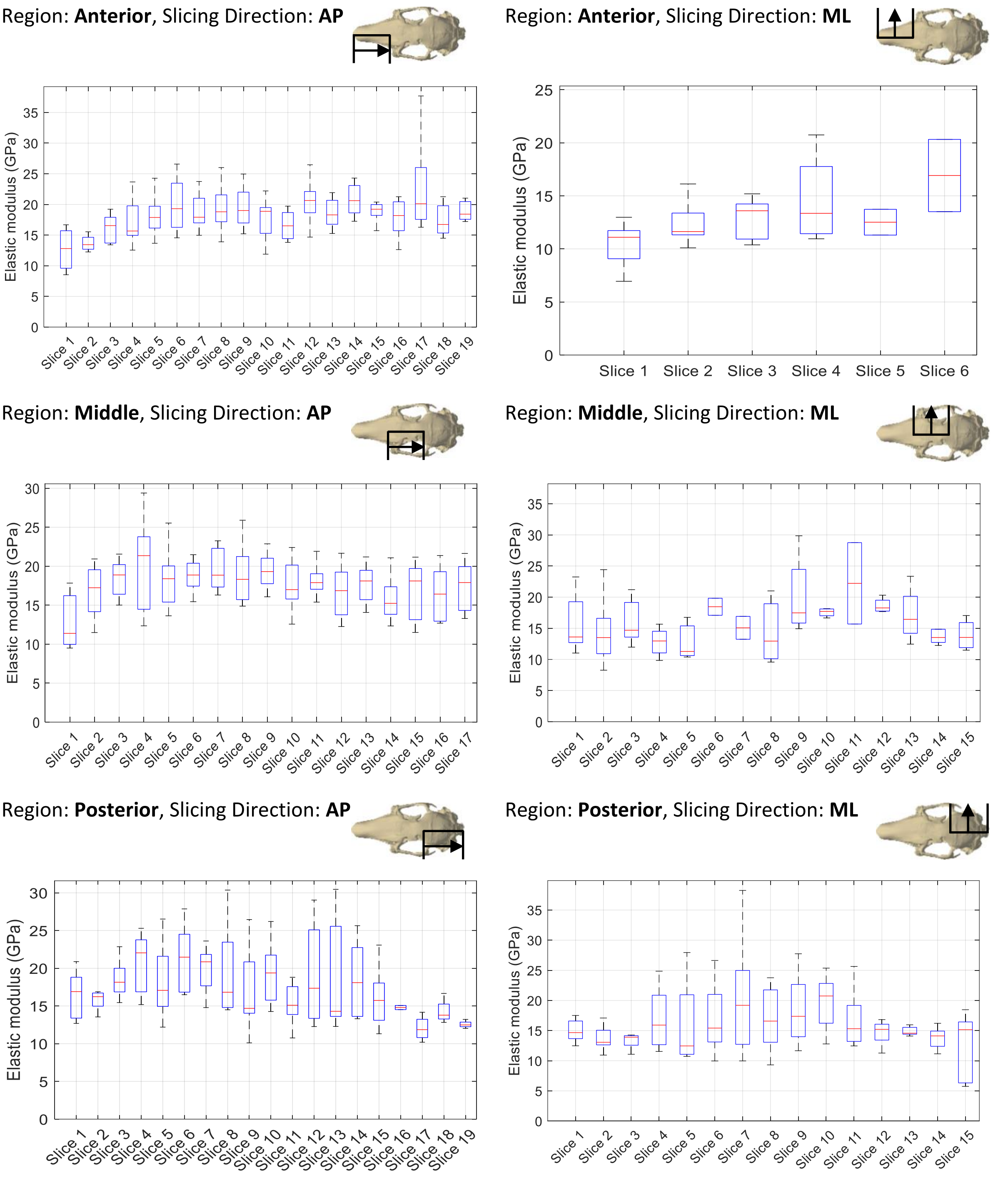


Figure S14. A box plot showing the minimum, first quartile, median, third quartile, and maximum elastic modulus measured in each slice of a region in Rabbit 2. The direction in which a region was analysed is defined as: anteroposterior (AP) and mediolateral (ML). Images accompanying each box plot illustrate the location of each region in the skull and the direction of the slicing.

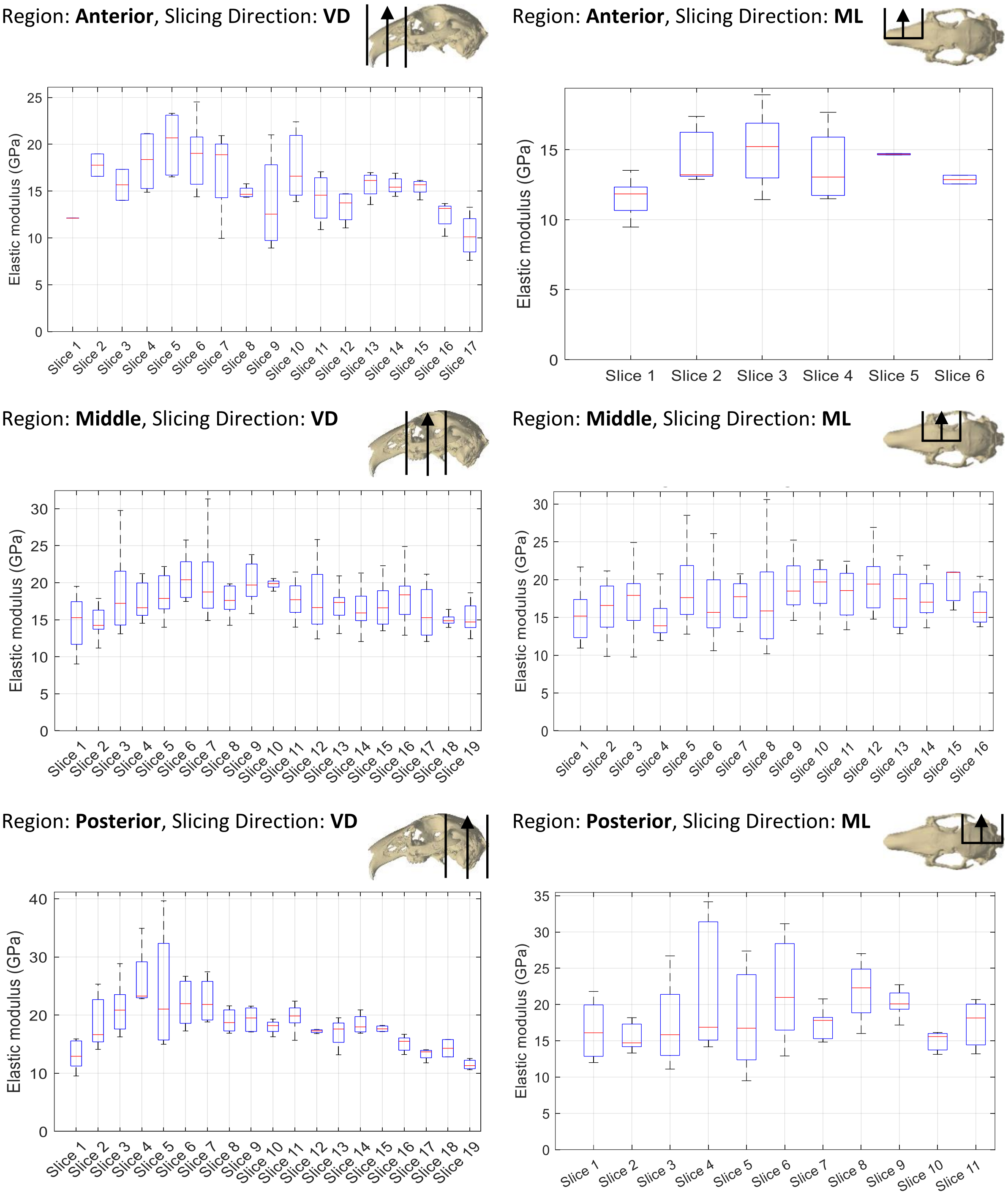


Figure S15. A box plot showing the minimum, first quartile, median, third quartile, and maximum elastic modulus measured in each slice of a region in Rabbit 3. The direction in which a region was analysed is defined as: ventrodorsal (VD) and mediolateral (ML). Images accompanying each box plot illustrate the location of each region in the skull and the direction of the slicing.
